# Alpha-catenin-Dependent Recruitment of the Centrosomal Protein CAP350 to Adherens Junctions Allows Epithelial Cells to Acquire a Columnar Shape

**DOI:** 10.1371/journal.pbio.1002087

**Published:** 2015-03-12

**Authors:** Maria P. Gavilan, Marina Arjona, Angel Zurbano, Etienne Formstecher, Juan R. Martinez-Morales, Michel Bornens, Rosa M. Rios

**Affiliations:** 1 Centro Andaluz de Biología Molecular y Medicina Regenerativa (CABIMER), CSIC, Sevilla, Spain; 2 Hybrigenics Services SA, Paris, France; 3 Centro Andaluz de Biología del Desarrollo, CSIC/UPO/JA, Sevilla, Spain; 4 UMR144 CNRS-Institut Curie, Paris, France; University of Virginia, UNITED STATES

## Abstract

Epithelial morphogenesis involves a dramatic reorganisation of the microtubule cytoskeleton. How this complex process is controlled at the molecular level is still largely unknown. Here, we report that the centrosomal microtubule (MT)-binding protein CAP350 localises at adherens junctions in epithelial cells. By two-hybrid screening, we identified a direct interaction of CAP350 with the adhesion protein α-catenin that was further confirmed by co-immunoprecipitation experiments. Block of epithelial cadherin (E-cadherin)-mediated cell-cell adhesion or α-catenin depletion prevented CAP350 localisation at cell-cell junctions. Knocking down junction-located CAP350 inhibited the establishment of an apico-basal array of microtubules and impaired the acquisition of columnar shape in Madin-Darby canine kidney II (MDCKII) cells grown as polarised epithelia. Furthermore, MDCKII cystogenesis was also defective in junctional CAP350-depleted cells. CAP350-depleted MDCKII cysts were smaller and contained either multiple lumens or no lumen. Membrane polarity was not affected, but cortical microtubule bundles did not properly form. Our results indicate that CAP350 may act as an adaptor between adherens junctions and microtubules, thus regulating epithelial differentiation and contributing to the definition of cell architecture. We also uncover a central role of α-catenin in global cytoskeleton remodelling, in which it acts not only on actin but also on MT reorganisation during epithelial morphogenesis.

## Abstract 2

In epithelial cells, the normally centrosomal protein CAP350 binds to α-catenin at adherens junctions and helps to establish the cells' parallel apico-basal microtubule array and columnar shape.

## Author Summary

Epithelia cover all the surfaces of and the cavities throughout the body and serve as barriers between the organism and its external environment. Epithelial differentiation requires the coordination in space and time of several mechanisms that ultimately lead to the acquisition of distinctive epithelial features, including apical-basal polarity, specialised cell-cell junctions, and columnar shape. Epithelial differentiation also induces the reorganisation of three cytoskeletal networks: actin filaments, intermediate filaments, and microtubules. In simple epithelia, cadherins and their cytoplasmic binding partners catenins play a crucial role in connecting cell-cell junctions to the actin cytoskeleton. The cadherin extracellular domain forms adhesive contacts between adjacent cells, and their cytoplasmic tail indirectly binds the actin-binding protein α-catenin, thus linking cell-cell junctions to the underlying actin cytoskeleton. We report here an additional role of α-catenin in remodelling microtubules during epithelial differentiation. In most epithelial cells, microtubules are organised as parallel bundles aligned along the apico-basal axis and as apical and basal plasma membrane-associated networks. We demonstrate that the microtubule-binding protein CAP350, which is only localised at the centrosome in most cells, is also recruited at cell–cell junctions in epithelial cells through its binding to α-catenin. In the absence of junctional CAP350, microtubules are unable to reorganise in bundles, and cells do not acquire columnar shape. Our results suggest that recruitment of centrosomal proteins to cell-cell junctions could be a general mechanism to control microtubule reorganisation in neighbour cells during epithelial differentiation.

## Introduction

Epithelial polarisation involves a coordinated series of events resulting in the asymmetric segregation of plasma membrane into apical and basolateral domains. The establishment of apico-basal polarity is also accompanied by asymmetric distribution of intracellular organelles, cytoskeleton reorganisation, and polarised membrane trafficking [1]. How the intracellular redistribution of organelles and the establishment of plasma membrane domains are coupled to define the spatial orientation of cell polarity is, however, not well understood.

The MT cytoskeleton undergoes a profound reorganisation during epithelial polarisation [2]. In many epithelia, MTs are prominently organised in bundles aligned along the apico-basal axis with their minus ends oriented toward the apical membrane and plus ends toward the basal membrane and as networks of mixed polarity underneath the apical and basal membranes. The molecular events underlying MT reorganisation and their contribution to epithelial morphogenesis remain unclear. In addition, these mechanisms seem to be different depending on the tissue. For instance, in the stratified epidermis, proliferative basal cells have a radial array of MTs organised around the centrosome (CTR), while differentiated cells have acentrosomal cortical MTs. During epidermal differentiation, desmosomes appear to be essential to organise MTs around the cell cortex. The centrosomal proteins Ninein, Lis1, and Ndel1 are recruited to desmosomes by desmoplakin [3–5], and both desmoplakin and Lis1 are required to organise MTs at the cortex. In simple epithelia, lateral intercellular junctions are largely composed of cadherin-based cell–cell contacts or adherens junctions (AJs). AJs consist of cadherin-adhesion receptors and associated cytoplasmic proteins, collectively called catenins. Cadherins form an adhesive interface all along the lateral domain but are also organised into special complexes known as zonula adherens (ZA) that are localised at the apical end. AJs are enriched in MTs, and MT bundles aligned along the apico-basal axis have their minus ends in close proximity to the ZA [6]. The molecular players underlying the AJ–MT connection remain, however, mostly unknown.

Although cadherins are known to cooperate with the actin cytoskeleton [7,8], a growing body of evidence also links cadherins to MTs. Indeed, it has been shown that MT disruption either by overall depolymerisation or by freezing the dynamic activity of MT plus ends perturbs cadherin-based cell-cell contacts [9]. Furthermore, the expression of exogenous cadherins in CTR-free cytoplasts increased the number of MTs [10]. This effect, which was dependent on the formation of cell–cell contacts, was mimicked by application of beads coated with stimulatory anti-cadherin antibody and suppressed by overexpression of the cytoplasmic cadherin tail [10]. Moreover, targeting α-catenin, but not p120-catenin or β-catenin, to the plasma membrane reproduced the MT-stabilising activity of E-cadherin in this assay [11,12]. In mammalian epithelial cells, a protein complex containing Plekha7, the minus-end MT-binding protein Nezha/CAMSAP-3/Patronin, and KIF3C was reported to be recruited to ZA by interaction of Plekha7 with p120-catenin [13]. In nonpolarised individual cells, CAMSAP-3 is localised at the CTR and at minus ends of noncentrosomal MTs [14]. Whether CAMSAP-3 regulates the orientation of MTs in polarised epithelial cells remains to be elucidated. p120-catenin was also shown to interact with the MT plus-end binding protein CLASP2 in progenitor epidermal cells, suggesting that, depending on the cell context, p120-catenin could recruit opposite MT-binding activities [15].

CAP350 is a large and highly conserved cytoskeleton-associated protein-glycine-rich (CAP-Gly) domain-containing protein that directly binds MTs through its N-terminal domain. It localises at the CTR, where it might play a role in MT anchoring [16,17]. CAP350 also participates in MT stabilisation at both the Golgi area and centrioles [17,18]. It was reported that CAP350 interacts with the centrosomal protein FOP and recruits it to the CTR [16]. Interestingly, a novel protein superfamily, the TON1 Recruiting Motif (TRM) protein family, has been identified in plants [19]. An archetypal member of this family, TRM1, is a MT-associated protein that localises to cortical MTs. TRM1 interacts in vivo with the TON1 protein that shares similarity with human FOP and is essential for MT organisation at the cortex [19]. Three motifs of TRMs (M3, M4, and M2) are found in CAP350. The M2 motif of CAP350 is responsible for FOP recruitment and was shown to interact with plant TON1 protein in yeast. These results suggest a conservation of CAP350-FOP centrosomal components in plant cells where they bind cortical MTs. CAP350 also targets the deubiquitinating enzyme cylindromatosis (CYLD) to the CTR in mammalian cells. This targeting is required for proper ciliogenesis, supporting an indirect role of CAP350 in primary cilium formation [20].

Studies on CAP350 have been generally performed in cells that do not make adhesive contacts with one another or do not express E-cadherin, such as Hela cells [16–18]. Intriguingly, a stable isotope labelling by amino acids in cell culture (SILAC)-mass spectrometry (MS)-based proteomic analysis of isolated adherent surfaces of epithelial MDCKII cells identified CAP350 as one of the most enriched cellular proteins [21]. This study suggested both the existence of a membrane-bound fraction of CAP350 in polarised kidney cells as well as a previously unknown role of this protein at the cell periphery.

In this work, we report that CAP350 localises at the cell cortex in a cadherin-adhesion-dependent manner. A two-hybrid screen for centrosomal protein partners revealed a consistent high-confidence interaction of CAP350 with the adhesion protein α-catenin. By using MDCKII cells grown either as polarised epithelia or as cysts, we show that CAP350 is required for epithelial morphogenesis.

## Results

### CAP350 Localises at Cell-Cell Contacts in Epithelial Cells

**Fig 1:**
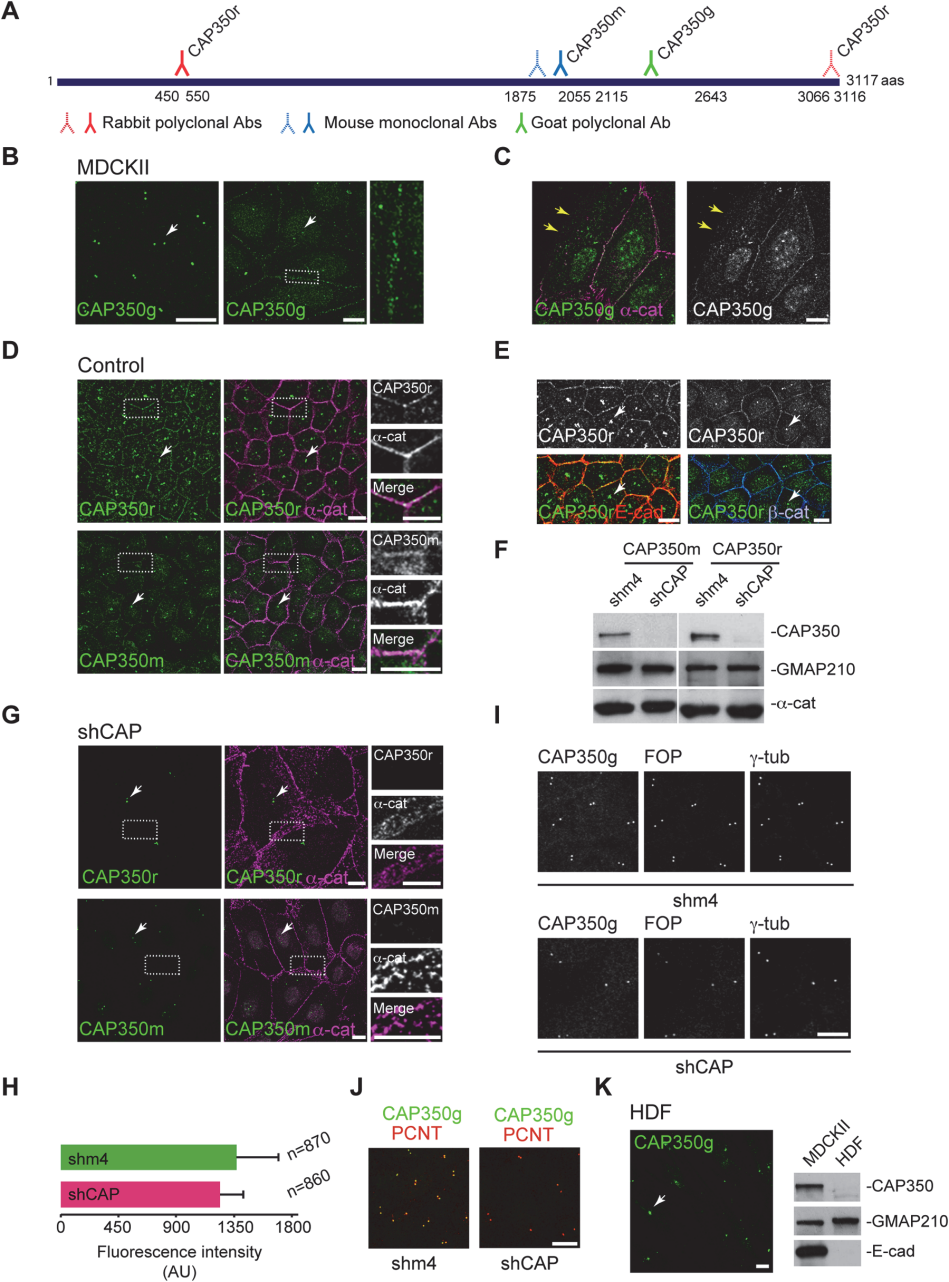
CAP350 is a cell-cell junction protein. (**A**) Diagram of CAP350 showing the different antigen recognition sites for all CAP350 antibodies used in this study. Continuous and dotted lines represent antibodies used for immunofluorescence (IF) and western blot (WB), respectively. (**B**) IF analysis of CAP350 localisation in either non-extracted (left) or Triton extracted (right) MDCKII cells using the anti-CAP350 goat antibody (CAP350g). Enlarged image of the outlined area is shown at right. Hereafter, white arrows indicate the CTR. (**C**) Merged image of control MDCKII cells double labelled for CAP350 and α-catenin. Note the co-localisation of both signals at cell-cell contacts and the absence of signals at the free edge (yellow arrows). Single labelling for CAP350 is also shown at right. (**D**) Control MDCKII cells processed by IF with either anti-CAP350 rabbit antibody (CAP350r, top panels) or anti-CAP350 mouse antibody (CAP350m, bottom) and α-catenin antibodies. At right, high-magnification images of the boxed areas showing single labellings and merged images. (**E**) Images of control MDCKII cells showing CAP350 co-localisation with E-cadherin and β-catenin, respectively. (**F**) Representative WB of MDCKII cells with CAP350m and CAP350r antibodies showing efficient depletion of CAP350 in cells infected with shCAP350 (shCAP) lentiviruses compared to those infected with control shm4 lentivirus. GMAP210 was used as a loading control. Alpha-catenin levels were also examined. (**G**) Same as in (D) but in shCAP lentivirus-infected cells. (**H**) Quantification of centrosomal CAP350 fluorescence intensity. Data represent mean ± standard deviation (SD) of three independent experiments. AU = arbitrary units. (**I**) Control (top) or CAP350-knockdown (bottom) MDCKII cells triple labelled for CAP350, FOP, and γ-tubulin. Single images are shown. (**J**) Same as in (I), but cells were stained for CAP350 and pericentrin (PCNT). (**K**) IF analysis showing the absence of peripheral CAP350 staining in human dermal fibroblasts (HDF) in which CAP350 is restricted to the centrosome (HDF, left). WB analysis confirming the absence of both E-cadherin and CAP350 signals in total extracts of HDF (right). Bars = 10 μm. The data used to make this figure are available in S1 Data.

By using a previously characterised goat polyclonal antibody (Fig. 1A, CAP350g), we found that in methanol- or paraformaldehyde (PFA)-fixed MDCKII epithelial cells, CAP350 is essentially detected at the CTR (Fig. 1B, left panel). However, when cells were extracted with Triton before fixation, an additional cell peripheral CAP350 labelling was observed (Fig. 1B, right panel). As shown in Fig. 1C, CAP350 clearly co-localised with α-catenin at cell-cell contacts but it was absent from cell-substrate adhesion sites (Fig. 1C, arrows). These results suggested that CAP350 accumulated at cell-cell adhesions. Similar results were also obtained in human epithelial MCF10A cells (S1A Fig.). To confirm this peripheral localisation, we performed additional immunofluorescence (IF) experiments by using two other antibodies (Fig. 1A): a rabbit polyclonal antibody recognising the N-terminal part of the protein (CAP350r, Novus Biologicals) and a mouse monoclonal antibody raised against the central part of the protein (CAP350m, this work). Both antibodies revealed a fraction of CAP350 co-localising with α-catenin at cell-cell junctions, which was more conspicuous when cells became fully polarised (Fig. 1D). Double labelling for CAP350 and the adhesion proteins E-cadherin or β-catenin further indicated that, in addition to the CTR, CAP350 localised at cell-cell adhesion sites in epithelial cells (Fig. 1E).

In order to verify the specificity of the peripheral labelling, we developed three short hairpin RNA (shRNA) lentiviruses targeting different CAP350 sequences (shCAP1, shCAP2, and shCAP3) and infected MDCKII cells. As controls, we generated lentiviruses containing either empty vectors or shRNAs against CAP350 but including four point mutations (shm4). Four days post-infection, cells infected with any of the shCAP-lentiviruses showed reduced CAP350 expression when compared with either noninfected or control lentivirus-infected cells, as revealed by WB (S1B Fig.). When cells were infected with a mix of the three lentiviruses (hereafter shCAP-lentiviruses), CAP350 was almost undetectable by western blot (WB) (Fig. 1F). Immunofluorescence experiments showed that under these conditions cortex-associated CAP350 fraction disappeared, thus confirming the specificity of the staining (Fig. 1G). Similar phenotypes were observed in cells infected with any of the lentiviruses (S1C Fig.). Since lentiviruses targeted different CAP350 sequences, the possibility that the observed phenotypes were due to off-target effects is very unlikely. Under these conditions, however, the CTR-associated fraction was still present in most cells, suggesting a slower turnover of the protein at this location (Fig. 1G and S1C Fig.). These partially depleted cells appeared enlarged and displayed disorganised cell–cell contacts (Fig. 1G and S1C Fig.). Similar results were obtained in MCF10A cells (S1D Fig.).

Seven days post-infection, CAP350 centrosomal fraction was also exhausted (S1E Fig.). In order to preserve the centrosomal function of CAP350, all our experiments were carried out in cells infected with shRNA lentiviruses for four days, after which CAP350 was still maintained at the CTR. Indeed, quantification of CAP350 fluorescence intensity of more than 800 cells proved similar amounts of centrosomal CAP350 in shCAP- and shm4-infected cells 4 d post-infection (Fig. 1H). To further assess the integrity of the CTR, the presence of specific centrosomal markers such as FOP, whose recruitment to the CTR is CAP350-dependent, γ-tubulin, and pericentrin (PCNT) was analysed in cells transduced with specific or control lentiviruses for four days. Once again, no significant differences in the distribution of any of these centrosomal proteins were observed (Fig. 1I and Fig. 1J). Altogether, these analyses indicated that the experimental conditions we had set up would allow us to investigate the role of CAP350 at the cell periphery without severely affecting its function at the CTR (see below for CTR functionality).

To further substantiate that CAP350 is a cell-cell junction protein in epithelial cells, we examined the distribution of CAP350 in cells lacking E-cadherin. As shown in Fig. 1K, in human dermal fibroblasts (HDF) CAP350 was exclusively detected at the CTR by IF and hardly detectable by WB in whole-cell extracts, as expected for a strict centrosomal protein. Similar results were obtained in the MCF10A-derived cell line NeuT that expresses a constitutively active form of the oncogene ERBB2/HER/neu (S1F Fig.). In these cells, loss of E-cadherin and cell-cell adhesion was accompanied by loss of CAP350 labelling at the cell periphery but not at the CTR. A significant reduction of CAP350 expression was also detected by WB (S1F Fig.). These results, and those by others [17], suggest that whereas CAP350 is ubiquitously localised at the CTR, the presence of CAP350 at cell-cell contacts is a feature of E-cadherin-expressing epithelial cells.

### Junctional Localisation of CAP350 Depends on Cadherin-Based Cell-Cell Adhesion

**Fig 2:**
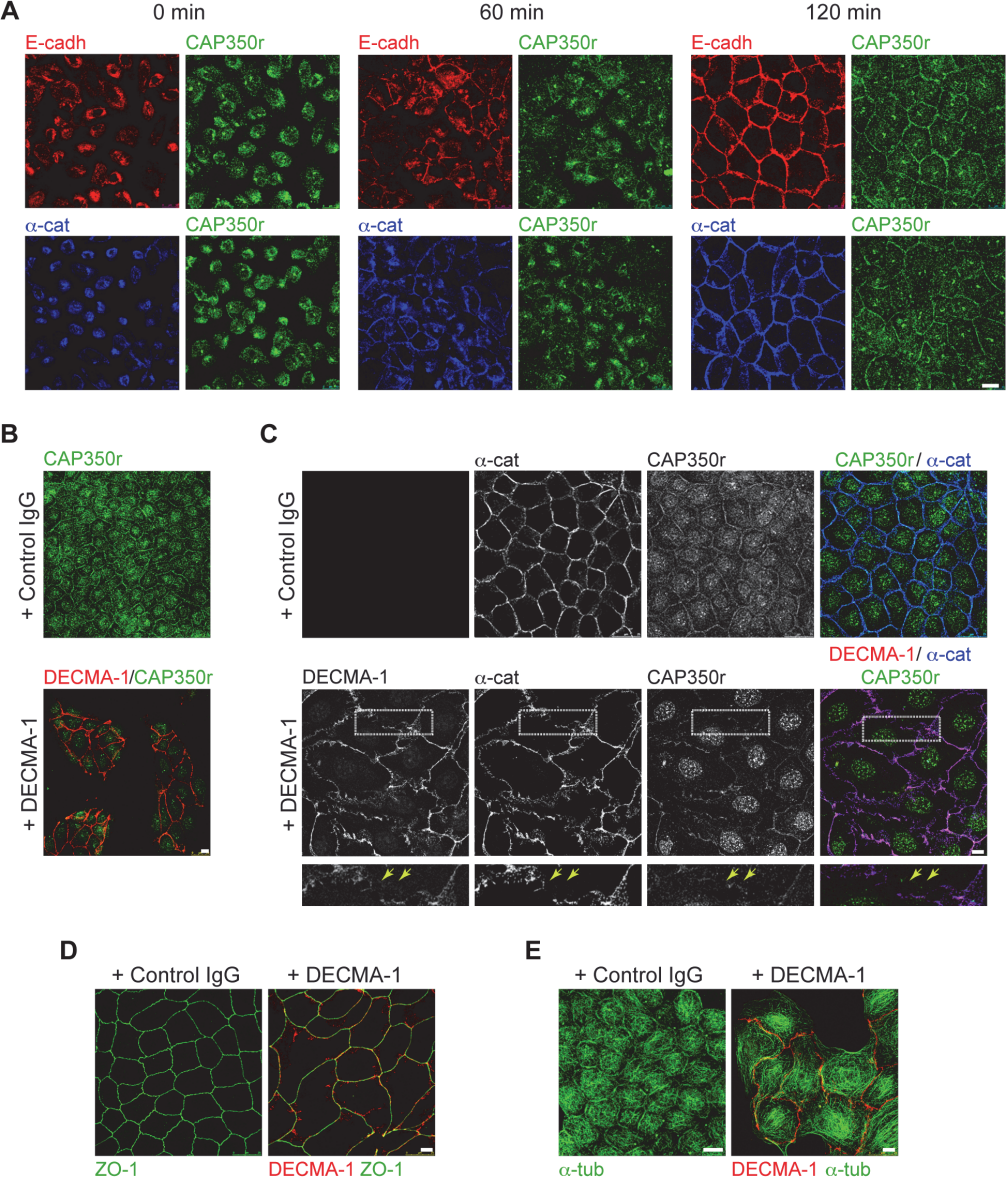
CAP350 peripheral labelling is dependent on E-cadherin-mediated cell adhesion. (**A**) Control cells were treated with 4 mM EGTA for 2 h to disrupt cell-cell contacts, fixed at the indicated times after washout, and stained with anti-E-cadherin and CAP350r (top) or anti-α-catenin and CAP350r (bottom) antibodies. Single labellings are shown. (**B**) Low-magnification images of MDCKII cells treated with either a control immunoglobulin G (IgG) antibody (Ab) or the E-cadherin blocking Ab (DECMA-1) for 72 h. Treated cells were then fixed and stained for CAP350. The presence of DECMA-1 antibody bound to E-cadherin at the cell surface was visualised with a secondary antibody. (**C**) Representative images of cells treated as in (B) and double labelled for α-catenin and CAP350. E-cadherin in DECMA-1-treated cells was revealed by incubation with a secondary antibody. Merged images are shown at right. High-magnification images of boxed areas are shown in the panels underneath. (**D**, **E**) MDCKII cells processed as in (B) but stained with zonula occludens protein 1 (ZO-1) (**D**) or α-tubulin antibodies (**E**). Bars = 10 μm.

To examine if the peripheral localisation of CAP350 depends on cell-cell adhesion, we first applied the calcium chelation method with ethylene glycol tetraacetic acid (EGTA) (Fig. 2A). As expected, under calcium chelation condition, cells detached from each other and rounded up. E-cadherin and α-catenin stainings disappeared from the plasma membrane (Fig. 2A, 0 min). Similarly, CAP350 peripheral labelling was abolished by EGTA treatment (Fig. 2A, 0 min). Calcium addition allowed de novo formation of cell-cell contacts. Sixty minutes after calcium addition, E-cadherin and α-catenin, but not CAP350, were detected at most of the cell-cell contacts (Fig. 2A, 60 min). By 2 h, CAP350 was also recruited to cell adhesions (Fig. 2A, 120 min).

Then, we wondered whether cadherin-based cell-cell adhesion was responsible for junctional CAP350 localisation. To address this issue, we specifically inhibited E-cadherin homophilic binding by using a blocking antibody against its extracellular domain DECMA-1 and examined the distribution of either E-cadherin, α-catenin, and CAP350. Cells were allowed to polarise for three days in the presence of either an irrelevant antibody (immunoglobulin G [IgG] control) or the DECMA-1 antibody. Contrary to the control, cells were unable to form confluent layers in the presence of DECMA-1 and appeared as isolated groups containing a variable number of cells (Fig. 2B). Cell morphology and cadherin-based contact integrity were also compromised. As shown in Fig. 2C, distribution patterns of either E-cadherin or α-catenin were altered or even abolished at some cell-cell contacts. Changes in CAP350 distribution completely paralleled those of E-cadherin or α-catenin (Fig. 2C). By contrast, the distribution of the zonula occludens protein 1 (ZO-1) remained intact (Fig. 2D), indicating that changes in CAP350 localisation were not due to nonspecific alterations at the plasma membrane. Remarkably, we observed severe modifications of MT network in the presence of the blocking antibody, pointing out the relevance of E-cadherin-based adhesion in the organisation of the MT cytoskeleton (Fig. 2E).

Taken together, these results indicate that during cell-cell adhesion formation, the centrosomal protein CAP350 localises to cell-cell contact sites and that this localisation is dependent of E-cadherin-mediated adhesion.

### CAP350 Interacts with α-catenin

**Fig 3:**
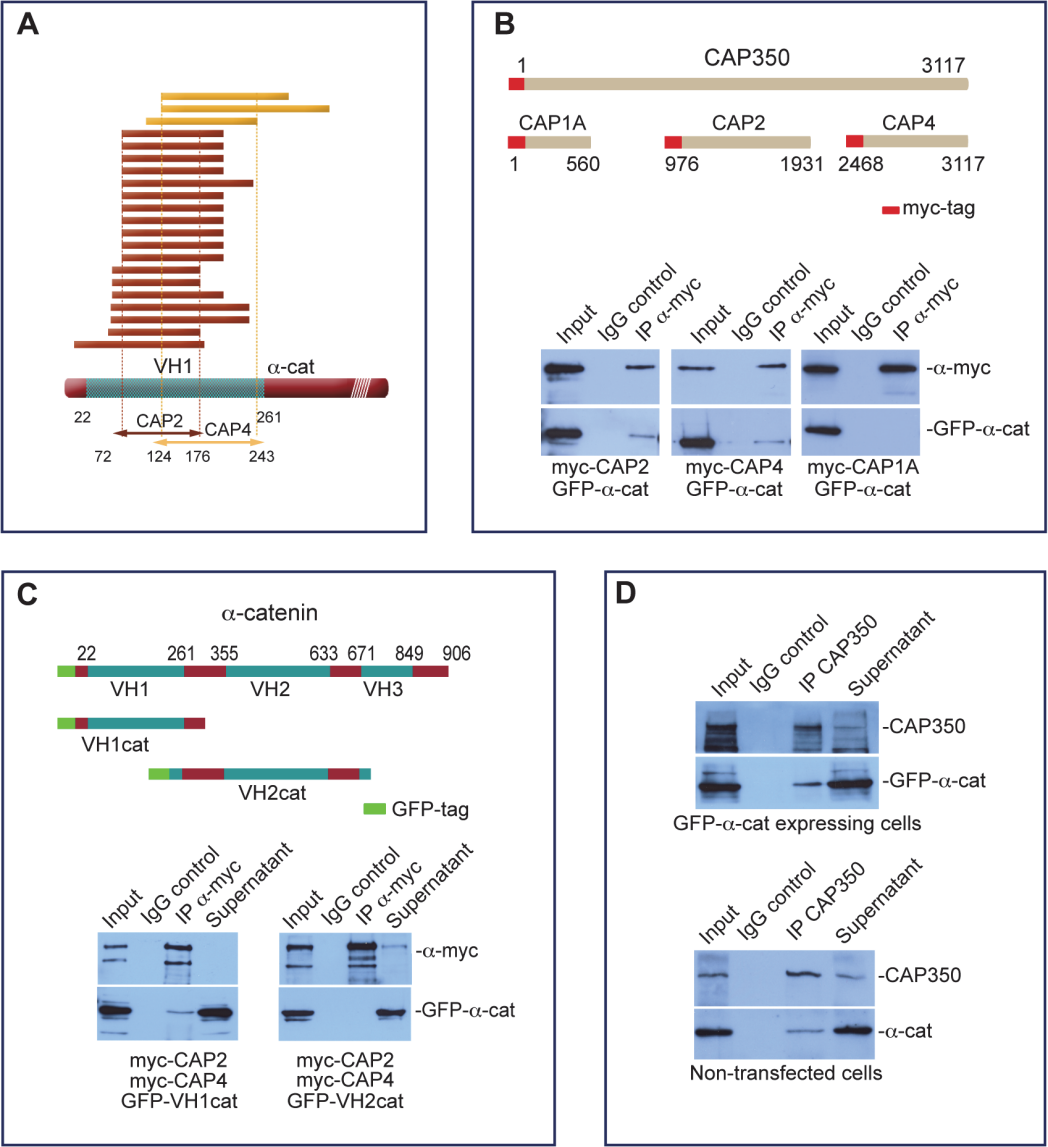
CAP350 directly interacts with α-catenin. (**A**) Summary of α-catenin clones isolated from a human PAZ-6 cell line complementary DNA (cDNA) library in a yeast two-hybrid screening, using as baits CAP2 and CAP4 fragments of CAP350. Eighteen positive clones (seven independent) of α-catenin were obtained when the CAP2 fragment was used as bait (dark red), whereas three positive clones were isolated with the bait fragment CAP4 (yellow). The mapped positions of clones are shown in amino acids. VH1: vinculin homology domain 1. (**B**) Schematic diagram of CAP350 showing the truncated mutants used in these assays and their relative positions. Hereafter, numbers represent amino acid positions in the full-length protein (top). A293T cells were double transfected with GFP-α-catenin and either myc-CAP1A, myc-CAP2, or myc-CAP4 truncated mutants. After IP with an anti-myc antibody, blots were revealed for GFP and myc (bottom). (**C**) Schematic representation of human α-catenin and the truncated mutants used in this work (top). Cells co-expressing myc-CAP2/myc-CAP4 and either GFP-VH1cat or GFP-VH2cat fragments were immunoprecipitated with an anti-myc antibody. Immunoprecipitates were then tested for the presence of myc-CAP2/myc-CAP4 or GFP (bottom). (**D**) Co-IP from GFP-α-catenin expressing cells using an anti-CAP350 antibody. Blots were revealed for CAP350 and GFP (top). Extracts from nontransfected MDCKII cells were incubated with anti-CAP350 antibody, and immunoprecipitates were analysed by WB for CAP350 and α-catenin (bottom).

To identify possible partners of CAP350, a two-hybrid screening was carried out using several fragments of the protein as baits. This screening identified a high-confidence interaction of CAP350 with α-catenin (Fig. 3A). Seven independent α-catenin clones were isolated with the CAP2 fragment (Fig. 3A, dark red), corresponding to the middle part of CAP350, and three with the CAP4 fragment (Fig. 3A, dark yellow), corresponding to the C-terminus of the protein. To confirm these interactions, we first performed co-immunoprecipitation (co-IP) experiments from A293T cells co-expressing a green fluorescent protein (GFP)-α-catenin fusion protein and myc-tagged versions of either CAP2 or CAP4 fragments of CAP350 (as represented in Fig. 3B, top panel). A CAP1A fragment was also included in the assay as a negative control. The results of these co-IP assays showed that GFP-α-catenin precipitated with both CAP2 and CAP4 fragments but not with the CAP1A fragment (Fig. 3B, bottom panel), suggesting that CAP350 contains two α-catenin binding sites.

All isolated clones contained a sequence that is included in the vinculin homology domain 1 (VH1) domain of the protein. To ascertain CAP350 binding to the VH1 domain of α-catenin, we generated two α-catenin-truncated mutants, VH1Cat consisting of the VH1 domain and VH2Cat roughly corresponding to the VH2 domain, to be used as a negative control (Fig. 3C, top panel). VH1 domain of α-catenin co-precipitated in the presence of both fragments, whereas no interaction was detected with the VH2Cat construct (Fig. 3C, bottom panels). Next, we used a lysate of GFP-α-catenin transfected cells to examine whether GFP-α-catenin co-immunoprecipitated with endogenous CAP350. As shown in Fig. 3D (top panel), anti-CAP350 antibodies co-immunoprecipitated both CAP350 and GFP-α-catenin. Finally, the presence of endogenous CAP350-α-catenin complexes was demonstrated by co-IP experiments in nontransfected MDCKII cells (Fig. 3D, bottom panel). These results reveal the existence of endogenous CAP350 and α-catenin complexes in MDCKII cells.

### CAP350 Is Recruited to Cell-Cell Contacts through Interaction with α-catenin

**Fig 4:**
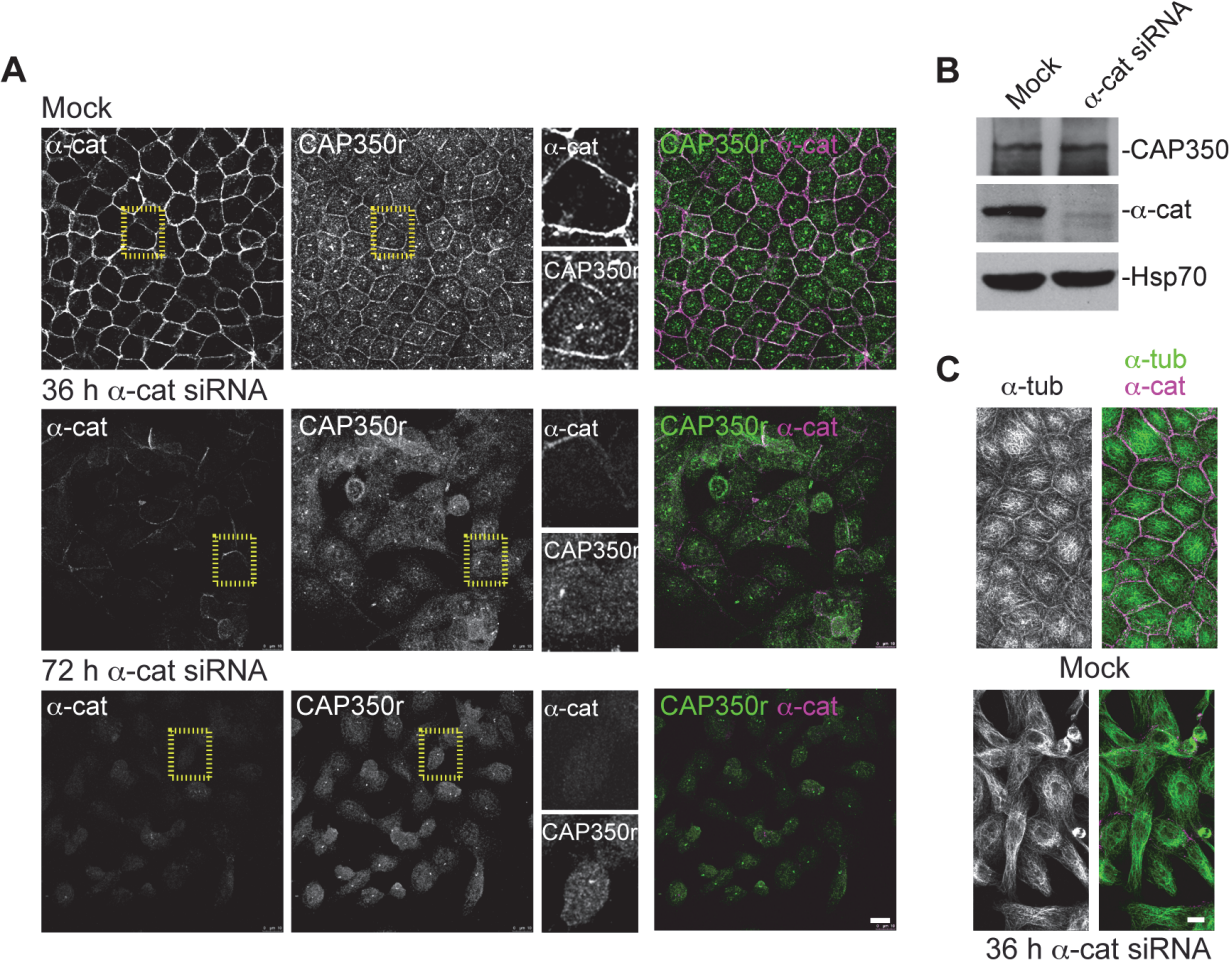
CAP350 localisation at the AJs depends on α-catenin. (**A**) MDCKII cells transfected with either a scramble (mock) or specific α-catenin siRNA were fixed 36 and 72 h post-transfection, processed for IF, and double stained with anti-α-catenin and CAP350r antibodies. Enlarged images of the outlined areas are shown at right. Merged images are shown at the far right. (**B**) Representative WB showing siRNA-mediated depletion of α-catenin in MDCKII cells. CAP350 levels were also examined, and HSP70 was included as a loading control. (**C**) IF staining of α-tubulin and α-catenin in control and α-catenin-depleted MDCKII cells, 36 h post-transfection. Bars = 10 μm.

To assess the contribution of α-catenin in CAP350 recruitment to cadherin-based cell-cell contacts, cells treated with small interfering RNA (siRNA) against α-catenin were examined by IF 36 h and 72 h after transfection (Fig. 4A). Thirty-six hours after transfection, cells began to detach from each other, but some cell-cell contacts still remained. Under these conditions, both junctional α-catenin and CAP350 stainings were significantly diminished, and only residual α-catenin-CAP350 co-localisation was detected at the remaining cell-cell contacts. Seventy-two hours after α-catenin siRNA transfection, when most cell-cell contacts were disrupted, CAP350 peripheral localisation was completely abolished. WB of α-catenin siRNA transfected cell extracts confirmed almost full depletion of α-catenin after 72 h (Fig. 4B). The amount of CAP350 in α-catenin-depleted cells did not change, indicating that disappearance of CAP350 peripheral labelling was not due to degradation (Fig. 4B). Interestingly, loss of α-catenin and subsequent dissociation of CAP350 resulted in the loss of the polarised pattern of MT cytoskeleton, further supporting the role of cadherin junctions in defining MT network architecture (Fig. 4C). These findings demonstrate that CAP350 is recruited to the AJs in an α-catenin-dependent manner.

### Ectopic CAP350 Is Able to Bundle MTs

**Fig 5:**
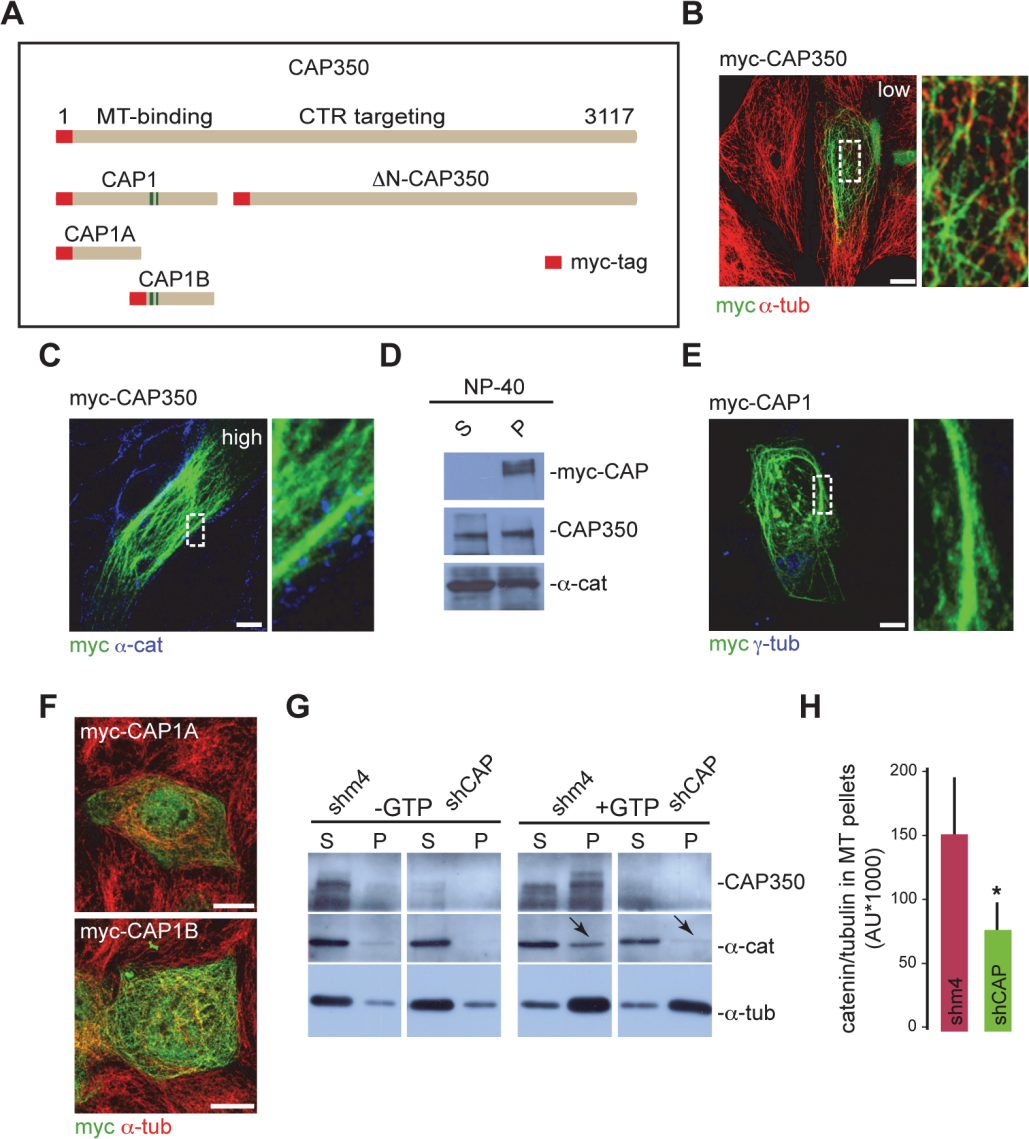
CAP350 targets α-catenin to MTs. (**A**) Diagram of CAP350 showing the additional truncated mutants used in this part of the study. All the truncated mutants were fused to a myc tag. (**B**) MDCKII cells expressing myc-CAP350 were double labelled for myc and α-tubulin. Merged image of a low-expressing cell is shown. At right, high-magnification image of the outlined area showing binding of myc-CAP350 along cytoplasmic MTs. (**C**) Merged image of a high-expressing myc-CAP350 cell double stained for myc and α-catenin. Magnification at right (taken from the boxed area) shows a cortical MT bundle. (**D**) WB of MDCKII cells expressing myc-CAP350 showing the distribution of myc-CAP350, CAP350, and α-catenin between NP-40-soluble (S) and insoluble (P) fractions. Alpha-catenin was used as a loading control. (**E**) MDCKII cells transfected with myc-CAP1 construct and labelled for myc and γ-tubulin. High-magnification image of a MT bundle is shown at right. (**F**) Images of myc-CAP1A (top) and myc-CAP1B (bottom) transfected cells double labelled for myc and α-tubulin. (**G**) MT-pelleting assay from control or CAP350-knockdown MDCKII cell lysates. After taxol-induced polimerisation and centrifugation through a sucrose cushion, supernatants (S) and pellets (P) were analysed by WB for CAP350, α-catenin, and α-tubulin. (**H**) Densitometric analysis of three representative WB. Bar graphs represent the ratio of α-catenin and α-tubulin in MT pellets from control or CAP350-knockdown cells. * *p* = 0.0342, two-tailed unpaired Student's *t* test. Bars = 10 μm. The data used to make this figure are available in S1 Data.

To characterise the molecular basis of CAP350 function in cell-cell adhesion, we ectopically expressed myc-CAP350 in MDCKII cells (Fig. 5A). As also reported in other cell types [17], at a low expression level CAP350 targeted the CTR (S2A Fig.). When the expression level increased, myc-CAP350 also associated with MTs, mostly in the pericentrosomal area (Fig. 5B). At a high expression level, the protein covered the whole MT network. Under these conditions, MTs did not arise from the CTR, and some unusually thick, bended MT bundles were observed (Fig. 5C), thus indicating that CAP350 is able to bind to the lattice of cytoplasmic MTs when present in excess. It must be noted that ectopic CAP350 was not detected at cell-cell contacts. Contrary to the endogenous protein, transfected myc-CAP350 was only detected in the insoluble fraction after detergent extraction (Fig. 5D) in agreement with the IF results. To investigate whether MT binding could prevent ectopic CAP350 targeting to cell-cell contacts, we expressed a truncated mutant lacking the MT-binding N-terminal domain but containing both α-catenin binding domains and the CTR targeting site (Fig. 5A). Unfortunately, results from these experiments were inconclusive since the truncated mutant was not detected either at cell-cell contacts or at the CTR, probably because of improper folding (S2B Fig.).

An even stronger MT-bundling effect was detected when a construct consisting of only the N-terminal MT-binding domain CAP1 was expressed (Fig. 5E). In vitro assays had previously shown that the N-terminal domain of CAP350 directly binds MTs through two independent regions [17]. To provide support to the MT-bundling activity of CAP1 domain, we generated two shorter N-terminal constructs (Fig. 5A) corresponding to the two independent MT-binding sites [17]. As shown in Fig. 5F, both truncated mutants were able to decorate MTs (although with different affinities), but none of them exhibited MT-bundling ability, indicating that these two domains have to form part of the same molecule to display this capacity. These experiments suggested that, in addition to its known MT-binding and stabilising properties, CAP350 might possess a genuine MT-bundling capacity.

### CAP350 Targets α-catenin to MTs In Vitro

Since CAP350 is able to bind both α-catenin and MTs, we then wondered whether CAP350 could recruit α-catenin to MTs. To answer this question, we carried out in vitro MT co-sedimentation assays. Soluble fractions from control or CAP350-depleted cells were incubated with taxol and assembled MTs sedimented by centrifugation through a sucrose cushion. As a negative control, guanosine-5’-triphosphate (GTP) and taxol were not added in parallel assays. As shown in Fig. 5G, α-catenin associated with MTs in a CAP350-dependent manner. Quantification of the ratio of α-catenin and tubulin in MT pellets from control- or CAP350-knockdown cells showed a 2.5-fold reduction of the amount of MT-bound α-catenin in the absence of CAP350 (Fig. 5H). Altogether, our results support a model in which CAP350 could bridge adhesion complexes at the plasma membrane to MTs: CAP350 would be recruited to the AJs by interaction between its CAP2 and CAP4 domains and the VH1 domain of α-catenin and, in turn, would bind MTs via its CAP1 domain (see below).

### CAP350 Is a Critical Determinant of the Columnar Architecture of MDCKII Cells

**Fig 6:**
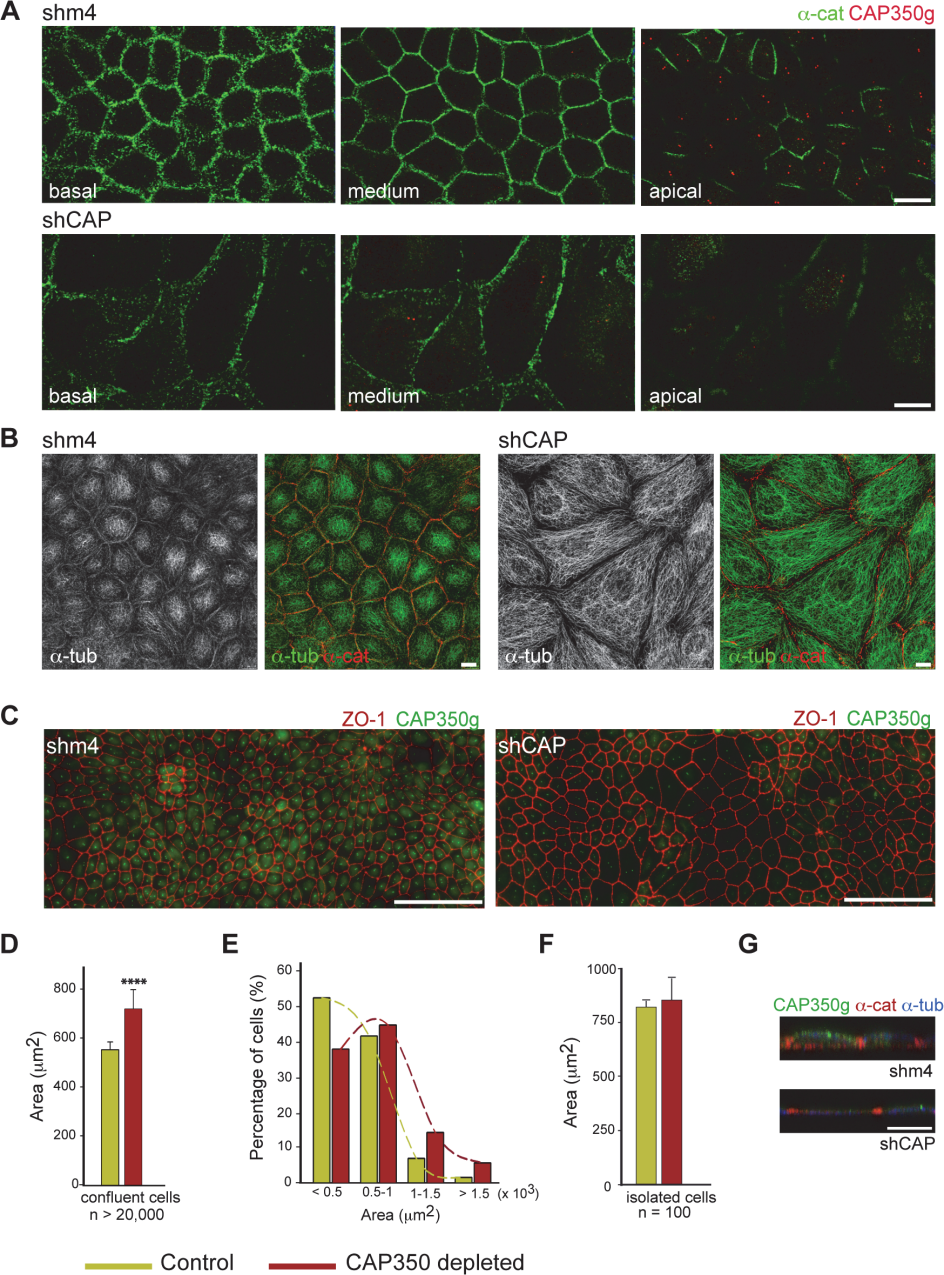
CAP350 Is Required for Apico-basal Polarisation. (**A**) Confocal Z-stack images of polarised MDCKII cells showing the distribution of α-catenin and CAP350. Sequential sections of either control (shm4, top) or CAP350-knockdown (shCAP, bottom) cells from basal to apical pole are shown. Bar = 10 μm. (**B**) Representative maximum projections of Z-stack images from either control (left) or CAP350-knockdown (right) cells stained for α-catenin and α-tubulin. Single labelling for α-tubulin and merged images are shown. (**C**) Mosaic images of polarised control (left) or CAP350-knockdown (right) MDCKII cells double labelled for ZO-1 and CAP350. Bar = 100 μm. (**D**) Quantification of the apical surface (μm^2^) enclosed by the ZO-1 signal in control (green) and CAP350-knockdown confluent cells (dark red). Bars represent mean values ± SD of three independent experiments (n > 20,000 for each case; **** *p* < 0.0001, two-tailed unpaired Student's *t* test). (**E**) Frequency plot showing the distribution of data represented in (D). (**F**) Quantification of the area covered by either control or CAP350-knockdown isolated cells. Data were collected from two duplicate experiments each conducted in triplicate and are represented as means ± SD. (**G**) XZ sections of control (top) and CAP350-knockdown (bottom) cells triple labelled for CAP350, α-catenin, and α-tubulin. Bar = 5 μm. The data used to make this figure are available in S1 Data.

In order to evaluate the role of CAP350 in epithelial morphogenesis, MDCKII cells were allowed to polarise for four days in the presence or in the absence of junctional CAP350. Cells transduced with control lentivirus showed a fully polarised phenotype including (i) defined AJs as revealed by staining for either α-catenin (Fig. 6A) or E-cadherin (S2C Fig.), (ii) an apically located CTR (Fig. 6A, top), and (iii) a MT-network with prominent cortical MTs (Fig. 6B, left). In contrast, in cells lacking junctional CAP350, AJs were strongly perturbed and became wide and undefined (Fig. 6A and S2C Fig.). The CTR remained close to the nucleus (Fig. 6A, bottom), and the apico-basal array of MTs was absent (Fig. 6B, right). Indeed, the MT network of CAP350-knockdown cells resembled that of nonpolarised cells. Tight junctions persisted under these conditions (see Fig. 6C), indicating a selective effect of CAP350 knockdown on AJs.

Strikingly, cells lacking junctional CAP350 appeared bigger and flatter than control cells (see also Fig. 1G). To quantify this phenotype, we measured the apical surface enclosed by the ZO-1 signal in mosaic images of confluent layers (Fig. 6C). Quantification of more than 20,000 cells revealed a 25% increase in the mean apical surface of knockdown cells (Fig. 6D). This percentage is probably underestimated since a number of cells may not be transduced. The distribution profile of cells ranked by apical area clearly showed a shift towards higher apical area values in depleted cells: the number of cells with the smallest area decreased, whereas the number of cells with highest area increased (Fig. 6E). To find out whether this increase in size is due to defective cell-cell adhesion or to increased cell-substrate adhesion, we also compared the surface occupied by control or depleted cells when they grew isolated. Data indicated no significant differences among them (Fig. 6F). Finally, fluorescence-activated cell-sorting (FACS) analysis revealed that the total volume of the cells remained unchanged after CAP350 depletion, suggesting that the increase in the apical area was accompanied by a decrease in height (S2D Fig.). Indeed, flattening of the cells was evident from vertical confocal images (Fig. 6G). Overall, these data demonstrate that CAP350 plays a key role in both AJ and MT-network reorganisation occurring during epithelial polarisation and, in this way, contributes to generate columnar epithelial cell architecture.

### CAP350 Depletion Affects Both AJ Formation and MT Network Reorganisation during Cell Polarisation

To gain further insights into CAP350 contribution to epithelial cell morphology, we performed live-imaging analysis of AJ formation and MT dynamics in control or CAP350-knockdown cells.

**Fig 7:**
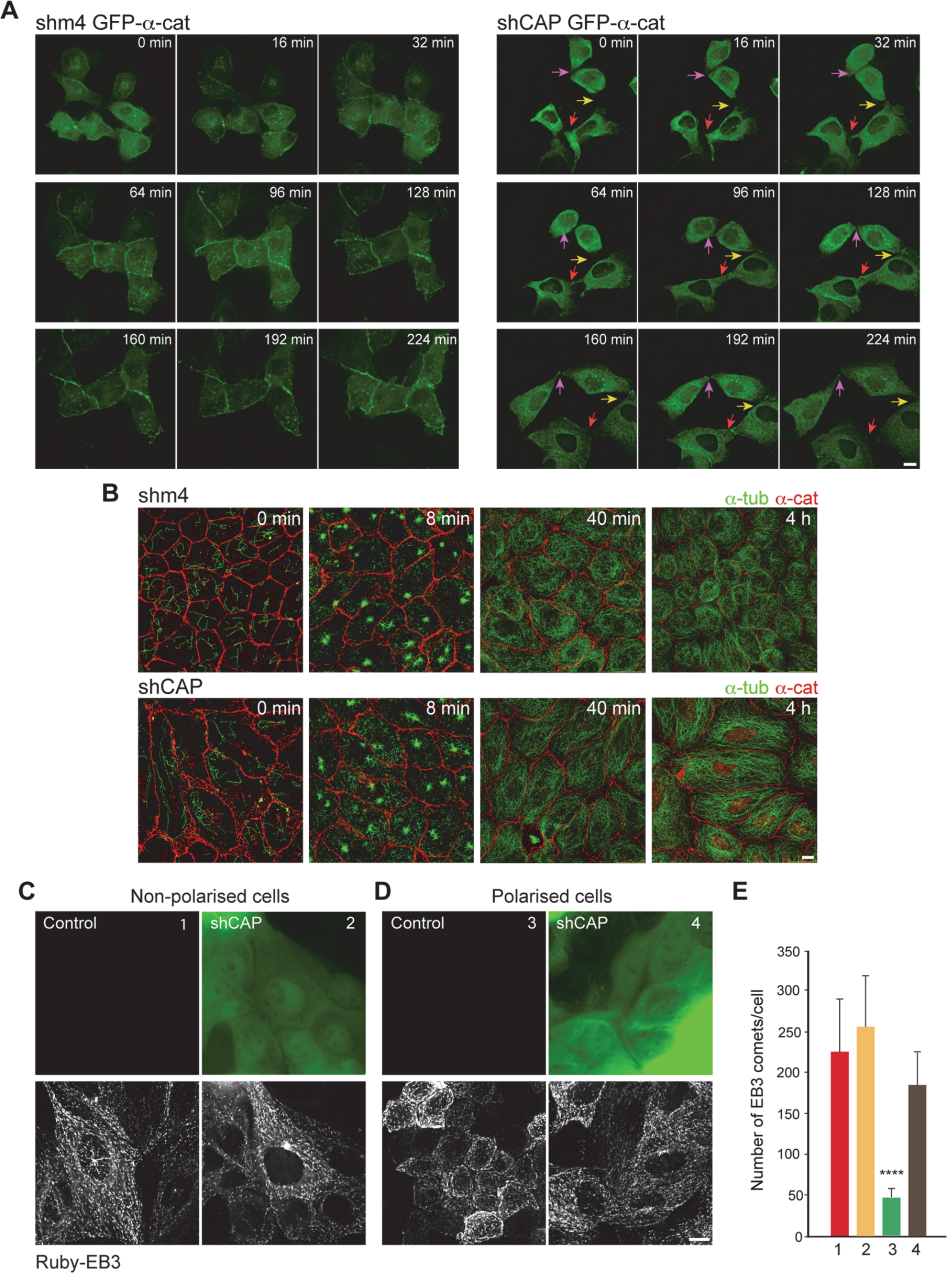
Defective cadherin-based cell-cell contact formation in the absence of junctional CAP350. (**A**) Time-lapse microscopy of MDCKII cells infected with either shm4 (left) or shCAP lentiviruses (right) and transfected with GFP-α-catenin. Cells were treated with 4 mM EGTA for 2 h to disrupt cell-cell contacts. EGTA was washed out and cells allowed recovery time in complete culture media. Time after EGTA removal is shown. Coloured arrows indicate representative examples of regions of unstable contact formation over time. (**B**) Control (top) or CAP350-knockdown (bottom) cells were treated with nocodazole for 2 h and incubated at 4°C for 30 min in the presence of the drug. At the indicated times after washout, cells were double labelled for α-catenin and α-tubulin. (**C**) Live-cell imaging of control and CAP350-knockdown subconfluent MDCKII cells, inducibly expressing Ruby-End binding protein 3 (EB3). Single images of GFP expression were taken at time 0 to confirm the effectiveness of shCAP viral transduction (upper panels). Maximal projections from the first four frames were overlaid to better visualise Ruby-EB3 tracks (lower panels). (**D**) Same as in (C), but cells were allowed to polarise for four days. Bars = 5 μm. (**E**) Quantification of the number of EB3 comets per cell under the conditions shown in (C) and (D). Bars represent mean values ± SD of three independent experiments (n > 10 for each case; **** *p* < 0.0001, one-way ANOVA followed by multiple comparisons test). The data used to make this figure are available in S1 Data.

First, we monitored calcium-induced AJ reassembly in GFP-α-catenin expressing cells infected with either control shm4 (Fig. 7A, left panels, and S1 Movie) or shCAP lentiviruses (Fig. 7A, right panels, and S2 Movie). Under calcium chelation, α-catenin dissociated from the plasma membrane (time 0). In cells expressing CAP350, 30 min after calcium addition α-catenin was already detected at the cell surface, and by 60 min contacts between cells were re-formed. In cells lacking junctional CAP350, α-catenin accumulated at spotlike junctions at the tips of cellular processes between neighbouring cells. However, these primordial contacts seemed to be unstable and disappeared. By 4 h, stable contacts between CAP350-knockdown cells were not yet established. Extended cell-cell contacts between depleted cells were clearly observed 7 h after calcium addition, although cells still occasionally detached from each other and appeared more mobile than control cells (S3A Fig., S3 Movie, and S4 Movie). Thus, the absence of junctional CAP350 delays the establishment of cell-cell contacts, probably by interfering with the ability of the cells to extend nascent cadherin-based adhesive contacts, and affects their stability.

Then, we performed MT regrowth experiments after nocodazole treatment in the control (Fig. 7B, top panels) and junctional CAP350-depleted cells (Fig. 7B, bottom panels) that had been allowed to polarise for four days. At 8 min after drug washout, prominent MTs were observed to emanate from the CTR under both conditions. No significant differences were detected either in density or length of CTR-growing MTs. These results indicated that not only the integrity but also MT nucleation activity at the CTR were preserved under our shRNA conditions. By 40 min a dense MT network occupying the whole cytoplasm had been formed in both the control and junctional CAP350-depleted cells. Four hours after drug washout, however, MT organisation was strikingly different: whereas MT network had acquired a polarised phenotype with conspicuous cortical MTs in control cells, the MT network of CAP350-knockdown cells resembled that of nonpolarised cells with long and mostly straight MTs.

In order to follow MT dynamics and/or growth during cell polarisation, we generated a tetracycline-inducible MDCKII cell line expressing the microtubule plus-end binding protein 3 (EB3) fused to the fluorescent protein Ruby (Ruby-EB3). Control or shCAP lentivirus-transduced Ruby-EB3 cells were recorded 12 h after tetracycline addition (Fig. 7C). Alternatively, they were allowed to polarise for four days before recording (Fig. 7D). In nonpolarised cells, both the distribution and the number of EB3 comets were similar either in the presence or in the absence of junctional CAP350 (Fig. 7C, Fig. 7E, S5 Movie, and S6 Movie). MT-nucleating activity of the CTR was also comparable in control or partial CAP350-knockdown cells. By contrast, the pattern of EB3 comet distribution dramatically changed in control cells after polarisation (Fig. 7D and S7 Movie). In fully polarised cells, CTR activity was hardly distinguishable and the EB3 comets decreased in number and acquired a cortical distribution (Fig. 7E). This reduction in centrosomal activity and MT dynamics is in agreement with the increase in MT stability that has been described in fully polarised cells [2,22]. In the absence of cortical CAP350, however, this shift of MT arrangement from a nonpolarised to a polarised state did not take place (Fig. 7D and S8 Movie). Indeed, the number of EB3 comets per cell was similar to that of control nonpolarised cells and four times higher than control polarised cells (Fig. 7E).

Taken together, these data support that CAP350 plays an important role in both the AJ formation and MT remodelling that occur during acquisition of columnar epithelial morphology. CAP350 thus emerges as a good candidate to mediate the reciprocal relationship between cadherin-based adhesion and MT cytoskeleton.

### Reduction of CAP350 Expression Levels Results in Defective Cystogenesis

**Fig 8:**
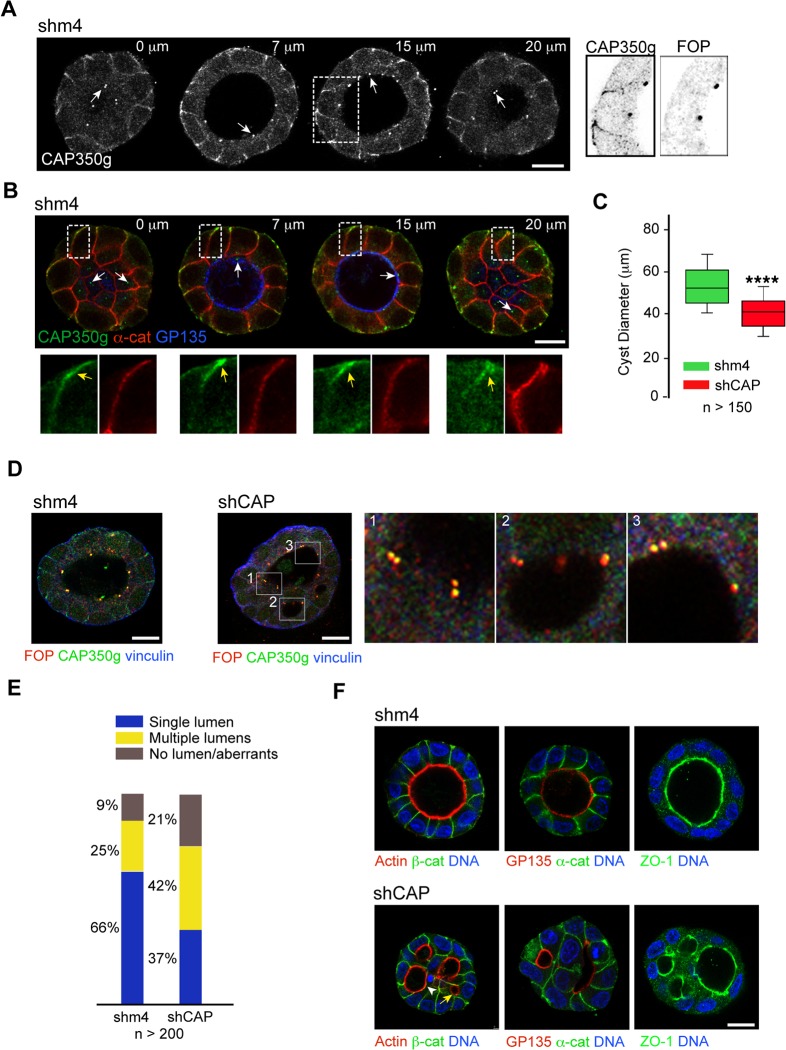
CAP350 knockdown leads to defective cystogenesis. (**A**) Sequential confocal z sections of a control MDCKII cyst in a 3-D culture from the top (left) to the middle (right) of the cyst. MDCKII cells were plated in matrigel for four days to allow cystogenesis, and cysts were fixed and double labelled for CAP350 and FOP. Single staining with CAP350g is shown. Magnifications at the right of the outlined area show inverted images of CAP350g and FOP labelling. (**B**) Images showing sequential cross sections of a representative MDCKII control cyst stained for GP135 (blue) as an apical marker, α-catenin (red) as a basolateral marker, and CAP350 (green). Enlarged views of the boxed areas are shown on the lower panels. Note increasing accumulation of CAP350 towards the basal pole of cells (yellow arrows). (**C**) Box-and-whisker plot showing quantification of diameter in control or CAP350-knockdown MDCKII cysts. Top and bottom end boxes represent 75th and 25th percentiles, and whiskers represent 90th and 10th percentiles. The black line within the box marks the median. **** *p* < 0.0001 (two-tailed unpaired Student's *t* test). Data were collected from three independent experiments. (**D**) Single confocal sections through the middle region of either control (shm4) or CAP350-knockdown (shCAP) cysts fixed at day 4 and labelled for FOP, CAP350, and vinculin. High magnifications of boxed areas are shown at the far-right panels as indicated. (**E**) Quantification of lumen formation in both control and CAP350-knockdown cysts. Data come from four independent experiments. (**F**) Single confocal sections through the middle region of either the control (top) or CAP350-knockdown (bottom) cysts labelled for β-catenin, α-catenin, GP135, and ZO-1 as indicated. Rhodamine-phalloidin was used to label filamentous actin (F-actin). DNA was counterstained with 4 ',6-diamino-2-fenilindol (DAPI). Bars = 10 μm. The data used to make this figure are available in S1 Data.

The effect of CAP350 depletion on epithelial architecture prompted us to study the function of CAP350 on cystogenesis. Consistent with 2-D-cultured cells, CAP350 staining was found at both the CTR, which localises at the apical pole, and the cell-cell junctions in MDCKII control cysts (Fig. 8A). In contrast, FOP exclusively localised at the CTR (magnifications in Fig. 8A). A more careful examination showed that CAP350 labelling intensity inversely correlated with the distance to the apical pole, and therefore, CAP350 overlapped with α-catenin mainly at the most basal half of lateral cell surfaces (Fig. 8B).

Knockdown of CAP350 profoundly affected the number, the size, and the morphology of MDCKII cysts. The number of cysts formed from the same number of control- or shCAP-transduced cells plated on Matrigel was reduced by about 50%. Measurement of the diameter of more than 150 cysts revealed a 20% reduction in CAP350-knockdown cyst size compared to control cysts (Fig. 8C). These figures could reflect individual differences in the reduction of CAP350 expression level. It should be noted that successfully formed cysts from shCAP-transduced cells lacked cortical CAP350 but retained CAP350 and FOP at the CTR, which maintained its apical localisation (Fig. 8D).

**Fig 9:**
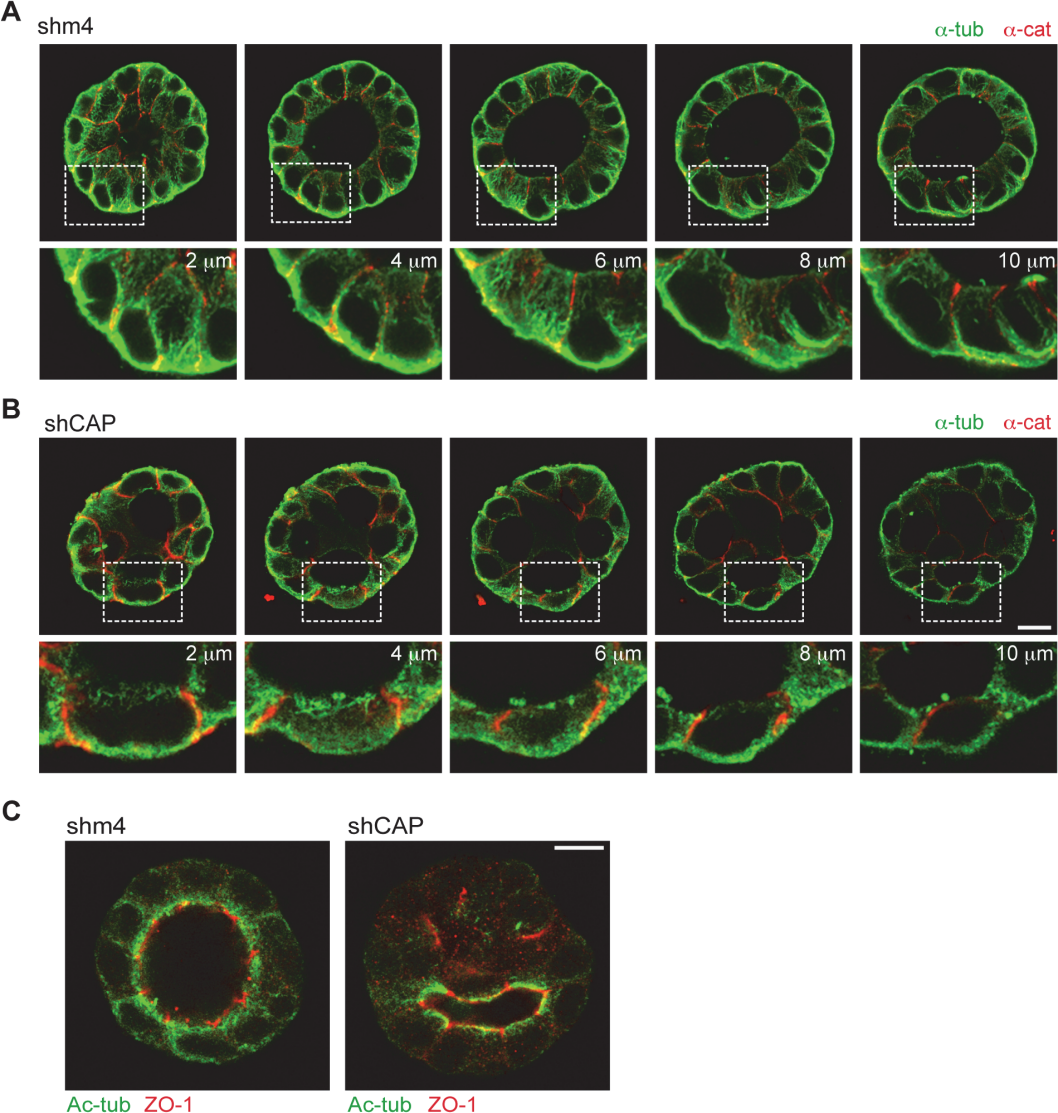
CAP350 depletion disrupts MT organisation during cystogenesis. (**A, B**) Sequential confocal sections of representative cysts formed from MDCKII cells infected with lentiviruses encoding either control shm4 (**A**) or shCAP (**B**) RNAs and stained for α-catenin and α-tubulin. Enlarged views of the boxed areas are shown at the bottom. (**C**) Single confocal section through the middle region of either control (left) or CAP350-knockdown (right) cysts fixed at day 4 and double labelled for acetylated-tubulin and ZO-1. Bars = 10 μm.

GP135/podocalyxin, an apical marker used to monitor lumen formation [23], showed that almost 70% of control cysts contained a single lumen (Fig. 8E). However, only 37% of CAP350-knockdown cysts were able to form a single lumen, while the rest contained multiple small lumens (42%) or consisted of cell aggregates without any obvious lumen (21%; Fig. 8D and Fig. 8E). Since defective cystogenesis can be attributed to cell polarity defects and subsequent mis-segregation of apical/basolateral membranes, we compared the localisation of several polarity markers in control and CAP350-knockdown cysts. In both cases, F-actin and GP135 were enriched at the apical membrane of all lumens, ZO-1 was restricted to the apical cell–cell contact region, and E-cadherin, α-catenin, and β-catenin were located at lateral cell–cell contacts. However, we could occasionally observe intracellular lumens (yellow arrows in Fig. 8F), co-localisation of α-catenin and β-catenin with apical markers (arrowheads in Fig. 8F), and cells containing two apical poles. Furthermore, cadherin-based cell-cell junctions appeared wider and disorganised in CAP350-knockdown cysts. We conclude that the global cell polarisation is taking place in CAP350-knockdown cysts, although a series of minor defects could be observed. Reorganisation of the MT cytoskeleton and the alignment of MTs along the apico-basal axis could be observed by confocal microscopy in the MDCKII cyst (Fig. 9A). In addition, a conspicuous MT network in the basal pole and a meshwork of short MTs at the apical pole of cells could be observed. Strikingly, cysts obtained from CAP350-knockdown cells contained less MTs. Both apical and basal MT networks persisted, but very few vertical MTs could be observed throughout the whole cyst (Fig. 9B). Acetylated tubulin labelling that usually lines the lumen under the F-actin ring maintained its distribution in CAP350-depleted cysts, further suggesting that the apical MT network is retained in CAP350-depleted cysts (Fig. 9C). These results demonstrate that CAP350 is required for organising the apico-basal arrangement of MTs in 3-D-cultured MDCKII cells and is therefore necessary for epithelial architecture.

### CAP350 Is Required for Epithelial Morphogenesis in Medaka Embryos

**Fig 10:**
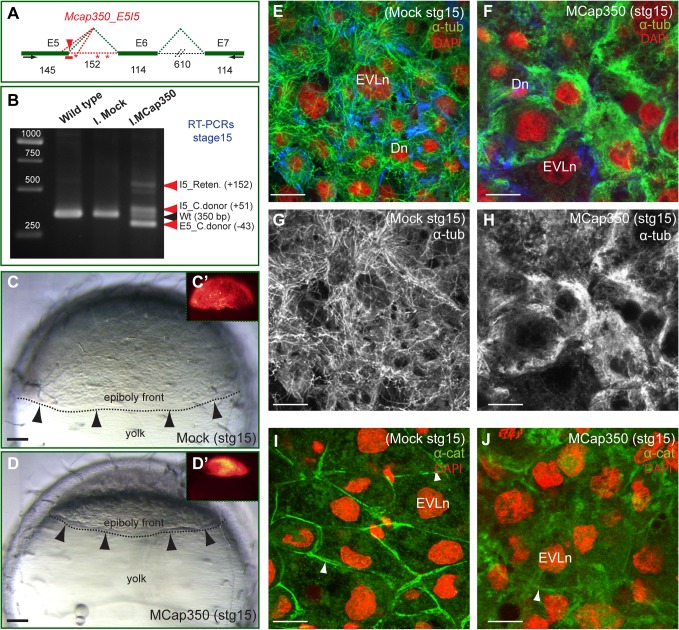
CAP350 is required for epithelial morphogenesis in medaka embryos. (**A**) Schematic representation of *cap350* exons 5–7 indicating the E5-I5 junction targeted by the morpholino Mcap350 (red arrowhead). Exon and intron sizes as well as intron retention and cryptic donor and acceptor sites are indicated by red dotted lines. (**B**) RT-PCRs show *cap350* aberrant splicing products in morpholino-injected embryos as compared with normal splicing (350 bp band) in wild-type and mock-injected embryos. PCR products derived from intron 5 retention (I5_Reten) and cryptic donors at exon 5 (E5_C.donor) and intron 5 (I5_C.donor) are indicated (red arrows). (**C–D**) Lateral views showing the progression of the epiboly front (arrow heads and dotted line) in Mock (**C**, **C'**) and Mcap350 (**D–D'**) injected embryos at stage 15. Fluorescent signal associated to the injected membrane-tagged tracer Lyn_tdTomato is shown in **C'** and **D'**. (**E**–**J**) Confocal microscopy analysis of α-tubulin (**E–H**) and α-catenin (**I**, **J**) whole mount immunostainings (green) in Mock and Mcap350 injected embryos at stage 15. Lyn_tdTomato (here in blue) injected embryos were also labelled with DAPI (here in red for simplicity). The nuclei of enveloping layer (EVLn) and deep cells (Dn) are indicated. Note the disorganized MT network (**F**, **H**) and the deficient recruitment of α-catenin to the cortex (**J**) in Mcap350-injected embryos. Bar = 100 μm (**C** and **D**) and 50 μm (**E–J**).

Finally, we aimed to understand the cellular role of CAP350 in the context of a developing organism. To this end, we employed morpholinos to knockdown *Cap350* gene expression in medaka fish (*Oryzias latipes*) embryos [24]. A splicing morpholino directed against the exon5-intron5 junction (MCap350) was designed and injected (150 μM) into two-cell medaka embryos (Fig. 10A). The ability of the morpholino to interfere with the splicing of the zygotic *Cap350* transcripts was assayed by reverse transcription polymerase chain reactions (RT-PCRs) in samples taken from wild-type as well as mock and MCap350-injected stage 15 embryos. Molecular analysis of morphant embryos showed a number of exon5-intron5 aberrant splicing events, which are consistent with the expected retention of intron 5 and the use of alternative (i.e., cryptic) donors of splicing at exon 5 and intron 5 (Fig. 10B). All the anomalous transcripts generated upon MCap350 interference include premature stop codons and hence yield exon-5 truncated forms of Cap350 (i.e., including only the first 340 N-terminal amino acids [aas]).

To evaluate the efficiency of the microinjections, the RNA of the membrane-tagged tracer Lyn_tdTomato was injected alone (mock injection) or co-injected with MCap350. Only those embryos expressing significant levels of Lyn_tdTomato at blastula stage (stage 11) were scored and analysed further. Strikingly, while epiboly progressed normally in mock-treated embryos at stage 15 (*n* = 56), in 100% of the MCap350-injected embryos (*n* = 76), the blastoderm cells failed to properly migrate and accumulated at the animal pole (Fig. 10C and Fig. 10D). Later during development, MCap350-injected embryos failed to gastrulate and degenerated before neurulation (stage 18). This observation indicates that CAP350 is required for embryo morphogenesis very early during development, shortly after the onset of zygotic transcription, and suggests that this loss-of-function phenotype corresponds to a severe cellular defect. In agreement with data obtained from MDCKII cells, both MT network and cell contacts, as revealed by α-tubulin and α-catenin immunostainings, appeared disorganised in morphant embryos (Fig. 10E–J).

## Discussion

Our findings identify CAP350 as an essential regulator of the MT architecture during epithelial differentiation. Recruitment of CAP350 to AJs by α-catenin confers to cells the capacity to develop apico-basal MT arrays and thus to acquire a columnar shape. Our data also indicate that, in addition to MT ends anchoring at the ZA, formation of cortical MTs would require MT stabilisation. Finally, they unveil a previously unknown role of α-catenin in coordinating actin and MT cytoskeleton remodelling during epithelial differentiation.

We show here that in E-cadherin-expressing cells CAP350 localised at both the CTR and AJs, whereas it was restricted to the CTR in nonepithelial cells or in cells that had lost E-cadherin expression because of oncogenic transformation. Interestingly, the total amount of CAP350 was also significantly reduced under these conditions, suggesting a coordinated regulation of either transcription or stability of both proteins. We also show that CAP350 recruitment to cell-cell contacts depends on both E-cadherin-based adhesion and α-catenin. On the other hand, CAP350-mediated binding of α-catenin to MTs could be demonstrated by MT-sedimentation experiments. Altogether, these results strongly support a role of CAP350 and α-catenin complexes at the AJ–MT interface. Remarkably, blocking of cadherin homotypic binding as well as depleting either α-catenin or junctional CAP350 led to similar perturbations on MT reorganisation during cell polarisation, highlighting the relevance of cadherin-based adhesion in MT regulation.

Biochemical approaches together with two-hybrid screening demonstrated that CAP350 could bind the N-terminal VH1 domain α-catenin directly. This domain has been reported to mediate α-catenin binding and α-catenin homodimerisation, which are mutually exclusive interactions [25–27]. Since the VH1 domain is engaged in α-catenin binding at the AJs, the question as to how CAP350 binds to these complexes is intriguing and remains unresolved. Several other proteins, including merlin, centralspindlin, Ajuba, and DLC1, have been also reported to interact with the VH1 domain of α-catenin, but the mechanism responsible for their targeting to the ZA has not been investigated further [28–30]. CAP350 contains two VH1-binding sites, suggesting that it may bind either two α-catenin monomers or one α-catenin homodimer. However, the asymmetric nature of the recently reported α-catenin dimer structure [31] suggests that CAP350 probably binds α-catenin monomers. Interestingly, a recent work has demonstrated that the complex formed by E-cadherin, β-catenin, and α-catenin is able to bind directly to F-actin under force application [32]. Anchoring of E-cadherin, β-catenin, and α-catenin complexes to actin filaments in polarised cells mainly occurs at the apical-basolateral boundary, where the actin ring develops, and probably involves a set of additional actin-binding or regulatory proteins [33]. We showed in the present work that CAP350 was absent from this region and instead co-localised with a fraction of basolateral E-cadherin, β-catenin, and α-catenin complexes. Indeed, whereas E-cadherin, β-catenin, and α-catenin stainings in MDCKII cells forming cysts extended from the apical ZA to the basal membrane, CAP350 localisation was restricted to the basal half of the lateral contacts, being excluded from the ZA. This suggests that, in addition to α-catenin, other, as-yet-unknown factors should collaborate to confer this differential localization. This spatial segregation also suggests that α-catenin may participate in different cadherin-based complexes located at specialised membrane regions, where it may perform specific functions: ZA-located α-catenin could regulate actin dynamics, whereas basolateral α-catenin could participate in MT reorganisation. Further supporting this view, junctional CAP350-depleted cells exhibited perturbed AJs but an apparently intact ZA.

Cell-cell junction formation and MT reorganisation are multistep and interdependent processes. Our data point out a role for CAP350 in several steps of the process. Recruitment of CAP350 to the cell surface occurred shortly after membrane contact formation between neighbour cells both in normal growth conditions and after calcium addition. The binding of α-catenin to E-cadherin and β-catenin complexes preceded CAP350 recruitment, thus suggesting that CAP350 binds to preassembled E-cadherin, β-catenin, and α-catenin complexes. Accordingly, CAP350 depletion did not prevent α-catenin association to cell-cell contacts nor did it induce its dissociation. However, our in vivo experiments demonstrated that CAP350 participates in the formation of AJs. This agrees with previous data showing that MTs are required for AJ assembly in tumour cell lines [9] and for AJ turnover in polarising epithelial cells in Drosophila [34]. A role for β-catenin-bound dynein in capturing MTs in developing cell-cell contacts has been reported [35]. It is worth keeping in mind that, in addition to its N-terminal MT-binding domain, CAP350 contains a CAP-Gly domain and two putative end binding protein 1 (EB1)-interacting sites that could mediate binding to MT plus ends. It could be speculated that two different complexes containing either β-catenin and dynein or α-catenin and CAP350 cooperate during AJ assembly by providing both tethering and stabilising activities.

Interestingly, α-catenin and CAP350 complex properties and behaviour clearly contrast with those of the p120-catenin, Pleka7, and CAMSAP-3 system [13]. CAMSAP-3 specifically localises at the ZA, whereas CAP350 localises at the basolateral membranes. In addition, CAMSAP-3 is recruited to cell junctions only in the mature cell-cell contacts and therefore appears to be more important for their maintenance than for their formation. While CAMSAP-3 may function by anchoring MT minus ends to the ZA once the apico-basal MT array has been established, its potential role in MT reorganisation during polarisation remains still to be investigated [14]. Taken together, this evidence suggests complementary roles for these two MT-regulatory systems during apico-basal polarisation. Alpha-catenin and CAP350 complexes would act earlier, regulating AJ formation and the concurrent transition from a radial mesenchymal array to an apico-basal epithelial architecture. In the latter stages of the differentiation process, recruitment of CAMSAP-3 and KIFC3 to ZA could serve to anchor MT minus ends at the ZA. Based on the perturbations induced by full CAP350 depletion on MT organisation, a role for CAP350 in MT-minus-end anchoring at the centrosome was proposed [16]. However, CAP350 also associated with MTs in the Golgi area [17], and its related plant protein TRM1 was found to bind to cortical MTs [19]. These results, together with the present work, argue against a restricted role in MT-minus-end anchoring. More work is necessary to clarify whether CAP350 is a specific MT-minus-end-binding protein or if it plays a more general role in MT dynamics.

The presence of two tandem MT-binding sites in the N-terminal domain of CAP350, both of which are required for its putative MT-bundling activity, provide mechanistic insights on how CAP350 could participate in the formation of apico-basal MT arrays. Interestingly, junctional CAP350-depleted cysts contained few cortical apico-basal MTs, whereas the apical and basal MT networks were preserved. This supports the hypothesis that junctional CAP350 depletion does not affect global MT dynamics but does affect the stabilisation of cortical MTs specifically. Indeed, junctional CAP350-depleted-cells exhibited a robust MT-nucleating activity at the CTR and were able to develop a dense MT network, as shown by Ruby-EB3 live-imaging experiments during MT regrowth. However, contrary to control cells, CAP350-depleted cells lacked cortical MTs. The absence of cortical MTs may contribute to defective lumen formation. Only a few studies about the contribution of MTs to spatial orientation of apico-basal polarity are available [36,37]. Notably, our results indicate that CAP350-dependent stabilisation of cortical MTs is not essential for segregation of apical and basolateral domains nor for CTR or Golgi Apparatus (GA) relocation to the apical pole, which is in agreement with the fact that these relocations take place before MT reorganisation is completed. Indeed, analysis of apical exocytosis in polarising MDCKII cells revealed that efficient secretion of apical proteins occurs well before completion of MT array rearrangement [38].

Strikingly, knockdown of CAP350 in medaka embryos blocked epiboly. Epiboly, the spreading of the blastoderm over the large yolk cell, is considered the first morphogenetic movement of the teleost embryo. Given that epiboly occurs shortly after the onset of zygotic transcription at stage 12 and that the morpholino only interferes with spliced zygotic transcripts, it is likely that CAP350 has an essential and general role in tissue morphogenesis. This early arrest of development in epiboly prevented further analysis of CAP350 during embryogenesis but allowed us to confirm in a developing organism the results obtained in polarised kidney cells. CAP350-depleted blastoderm cells exhibited defects in cell-cell adhesion and MT organisation. These defects can account for the block in epibolic movement, since both E-cadherin and MTs have been shown to be essential for epiboly; however, the underlying molecular mechanism remains to be elucidated.

In conclusion, this work reveals an essential role of the CAP350-α-catenin complex in defining the polarised columnar architecture of epithelial cells. Epithelial polarity and tissue architecture are compromised at early stages of epithelial-to-mesenchymal transition, a critical step in carcinoma progression and metastasis. Whether the CAP350-α-catenin complex somehow participates in this transition deserves further analysis.

## Materials and Methods

### Ethics Statement

Medaka experiments were performed in accordance with the guidelines of the “Comité de Ética CSIC”.

### Cells and Cyst Culture

MDCKII cells were cultured in MEM containing 10% FBS or grown in 3-D Matrigel cultures (BD), as previously described [39]. Briefly, cells were trypsinized to a single suspension at 5 x 10^3^ cells/ml in complete medium containing 2% Matrigel. Suspensions were plated into chamber glass slides (BD Falcon) precoated with 100% Matrigel. MDCK cysts were grown for four days, and the medium was renewed every two days. Human embryonic kidney A293T cells were cultured in DMEM supplemented with 10% FBS, l-glutamine, and penicillin-streptomycin. MCF10A and the MCF10A-derived cell line NeuT were grown in DMEM/F12 medium supplemented with 5% horse serum, 0.5 μg/ml hydrocortisone, 10 μg/ml insulin, 100 ng/ml cholera toxin, 20 ng/ml epidermal growth factor, l-glutamine, and penicillin-streptomycin. HDFs were grown in basal medium supplemented with 10% FBS, Fibroblast Growth Supplement (FGS, Innoprot), and penicillin-streptomycin. All cells were maintained in a 5% CO_2_ humidified incubator at 37°C.

### Yeast Two-Hybrid Screening

Yeast two-hybrid screening was performed on a random-primed cDNA library from human PAZ-6 cell line. The library was transformed into the Y187 yeast strain. The cDNAs encoding N-terminal (1–983 aa), C-terminal (2590–3118 aa), or internal (985–1929 aa) regions of hCAP350 were inserted into the pB27 bait plasmid. Forty-five, 87, and 46 million interactions were tested, and 294, 322, and 357 positive clones, respectively, were picked and analysed. The corresponding prey fragments were amplified by PCR and sequenced at their 5ʹ and 3ʹ junctions. These sequences were used to identify the corresponding gene in the GenBank database (National Center for Biotechnology Information) using a fully automated procedure.

### Reagents and Antibodies

Cells were treated with 4 mM EGTA (Sigma-Aldrich) for 2 h or 10 M Nocodazole (Sigma-Aldrich) for 3 h. Monoclonal anti-α-tubulin, anti-γ-tubulin, anti-acetylated-tubulin, anti-vinculin anti-c-myc, rat monoclonal anti-E-cadherin (DECMA-1), rabbit polyclonal anti-α-catenin, anti-β-catenin, and anti-α-tubulin antibodies were purchased from Sigma-Aldrich. Mouse monoclonal anti-α-catenin was obtained from Santa Cruz Biotechnology. Mouse monoclonal anti-E-cadherin and anti-β-catenin antibodies were from BD Biosciences. Rabbit polyclonal anti-ZO-1 was from Invitrogen. Rabbit polyclonal anti-GFP and anti-myc were purchased from ICL. Mouse monoclonal anti-GFP was purchased from Roche. Rabbit polyclonal anti-pericentrin was purchased from Covance. Mouse monoclonal anti-Hsp70 antibody was obtained from Abcam. Rabbit polyclonal anti-CAP350 antibodies (450–500 and 3066–3116 aa) were from Novus Biologicals. Rhodamine-phalloidin used to label F-actin was purchased from Sigma-Aldrich. Rabbit anti-GMAP210 has been previously characterised [40]. Goat polyclonal anti-CAP350 and rabbit polyclonal anti-FOP were kindly provided by E. Nigg (Biozentrum, University of Basel, Switzerland). Rabbit anti-GM130 and mouse anti-GP135 were kind gifts from Y. Misumi (Fukuoka University, Japan) and G. Ojakian (New York, United States). All secondary antibodies conjugated to DyLight fluorophores were from Jackson ImmunoResearch. Anti-IgG peroxidase-labelled secondary antibodies were from Amersham.

To generate CAP350 monoclonal antibodies, a construct containing amino acids 1875–2055 of human CAP350 was inserted into the expression vector pET28a (+), expressed in *Escherichia coli* strain BL21, and affinity purified using HIS-Select Nickel Affinity Gel according to the manufacturer's protocol. CAP350 monoclonal antibodies were generated by Protein Tools Unit, CNB/CSIC (Madrid, Spain).

### Lentiviral shRNAs

Stable RNAi was achieved by lentiviral shRNA. RNAi sequence against human CAP350 was 5ʹ-GTTACTCAGATGAACGATA-3ʹ. RNAi sequences against canine CAP350 (shCAP1 5ʹ-TTAAGAAGCAACCTGGAACAGTTGA-3ʹ, shCAP-2 5ʹ-GCAGCAAGAGAAGGCAGAAATTAAA-3ʹ, and shCAP3 5ʹ-AAGAGATGGAGCTAATTTCTTTGTG-3ʹ) were designed by using the BLOCK-iT RNAi Designer (Invitrogen). As controls, a shRNA containing four point mutations was used. Sequences were submitted to BLAST search to ensure targeting specificity. Complimentary oligonucleotides containing the shRNA sequences were synthesized with BglII and HindIII overhangs and were purchased from Sigma-Proligo. Oligonucleotides were annealed and cloned into pSUPER. Next, the H1 polymerase promoter-shRNA sequence cassette was removed with EcoRI/ClaI and ligated into the lentiviral vector pLVTHM (Addgene plasmid 12247). Two different versions of each lentivirus were generated: +GFP and −GFP. All inserts were sequenced. Experiments in MDCKII cells were carried out by infection with a mix of the three lentiviruses (named shCAP), unless otherwise indicated.

Virus production was performed according to standard protocols. 293T cells were plated the day before transfection to ensure them to be in the exponential growth phase at the moment of transfection. The pLVTHM vector, the packaging (psPAX2) plasmid, and the envelope plasmid (pMD2.G) (proportion 3:2:1) were co-transfected, and after 48 h and 72 h, viral supernatants were collected, combined, filtered through 0.45 μm PVDF filter (Fisher Scientific), and concentrated by ultracentrifugation (Beckman Coulter).

### Cell extraction, WB, IP, and MT-Pelleting Assays

For biochemical experiments, cells were extracted by incubating with 0.02% saponin and 0.02% BSA or 1% Triton X-100 in Pipes-Hepes-EGTA-MgCl_2_ (PHEM) buffer containing protease inhibitors for 5 min at room temperature (RT). Soluble fractions were centrifuged at 16,000 g for 20 min and supernatant collected. Insoluble fractions were solubilized with Laemmli sample buffer. SDS-PAGE, WB, IPs, and MT-pelleting assays were performed as described [41]. Densitometric analysis was performed on scanned images using Image Quant 5.2 software (GE Healthcare).

### Subcloning, Transfection, and siRNA

All CAP350 fragments (1–900, 1–560, 555–900, 976–1931, 2468–3117 aas) were obtained by PCR using pCS2-MT-CAP350 full length as template and EcoRI and XhoI restriction sites onto their 5ʹ and 3ʹ respectively to allow subclone into pCS2-MT 6 Myc vector. To generate ΔNCAP350 (976–3117) a fragment corresponding to 1720–3117 aas was obtained by PCR introducing StuI and XhoI restriction sites and by using pCS2-MT-CAP350 full length as template. Subsequently, pCS1-MT-CAP2 (976–1931) was digested with StuI and XhoI and the resulting vector was isolated and fused to 1720–3117 fragment. peGFP-CEP1 (1–900 aa) was generated by PCR by introducing EcoRI and BamHI sites. peGFP-α-catenin was a gift from Prof. J. Nelson. Both constructs of α-catenin (1–291 and 219–730 aa) were obtained by PCR introducing EcoRI and SalI sites and subcloned into peGFP-C2. Cells were transfected with either Lipofectamine2000 or by using Neon Transfection System (Invitrogen) according to the manufacturer's instructions.

To deplete α-catenin in MDCKII cells, we used the following siRNA: 5ʹ AUAACCUGAGGACAGAGGGCUUCUA 3ʹ. Scrambled siRNA was used as the control. Duplexes were obtained from Life Technologies and Sigma-Aldrich. siRNA transfection was performed with Neon Transfection System (Invitrogen) by following instructions from the supplier. Assays were performed 36 h and 72 h after transfection.

### FACS Analysis

Cell size analysis was done by flow cytometry. Measures were performed on a BD FACSCalibur flow cytometer (Becton Dickinson, US). Forward scatter height (FSC-H) was used as a measure of the cell size and was determined in MDCKII cells expressing either control or CAP350 shRNAs.

### Immunofluorescence

For immunofluorescence experiments, cells were grown on coverslips and fixed in either 100% methanol at −20°C for 6 min or 4% paraformaldehyde for 10 min at RT and permeabilised with 0.5% Triton X-100. Alternatively, cells were extracted with 0.3% Triton X-100 for 30 s at 37°C before fixation. Then, cells were incubated with primary antibodies for 1 h at RT, washed with 0.1% PBS-Tween, and incubated with the appropriate fluorescent secondary antibody for 40 min. Nuclei were counterstained with DAPI (1 μg/ml) after secondary antibody labelling.

MDCKII cysts were processed for immunofluorescence as follows: cysts were rinsed twice with DPBS (Sigma) containing CaCl_2_ and MgCl_2_, fixed with shaking for 30 min with 4% paraformaldehyde in DPBS, and permeabilised for 15 min with 0.5% triton in DPBS. To improve the staining of some centrosomal proteins, samples were treated with 0.5% SDS in DPBS for 10 min. After washing, nonspecific binding sites were blocked by rocking for 30 min in DPBS, 0.025% saponin, and 3% BSA. Samples were incubated overnight with primary antibodies at 4°C. Cysts were then rinsed three times with blocking buffer and incubated with secondary antibodies conjugated to DyLight fluorophores for 90 min at RT. Nuclei were counterstained with DAPI. Finally, samples were extensively washed with blocking buffer and DPBS and mounted in ProLong (Molecular Probes).

Stage 15 embryos were fixed in 4% paraformaldehyde in PBS overnight at 4°C. After chorion removal, embryos were washed in PBS and blocked for 2 h in PBS containing 0.2% Tween (PBT), 1% DMSO, and 10% FCS (blocking solution). Whole embryos were incubated overnight at 4°C with primary antibodies diluted in blocking solution. After washing in PBT, samples were incubated with Alexa-conjugated (Molecular Probes) secondary antibodies for 3 h at RT. After final rinses, embryos were counterstained with DAPI (5 μg/ml) and mounted with PBS/glycerol.

### MDCKII Ruby-EB3-Inducible Cell Line Generation

A Ruby-EB3-inducible cell line was generated using the Flp-In T-REx system (Invitrogen) according to the manufacturer's instructions. Briefly, a MDCKII host cell line was obtained in a two-step procedure. In the first step, MDCKII cells were transfected with pFRT/lacZeo2 followed by selection with 500 μg/ml Zeocin (Invitrogen). Single-copy integrants were identified by Southern blot analysis and β-galactosidase expression was analysed to identify clones with integration of the pFRT/LacZeo2 plasmid into a high-expression site (β-Gal Staining Kit, Invitrogen). In a second step, MDCKII cells were transfected with pcDNA6/TR and selected with 10 μg/ml Blasticidin (InvivoGen) until colonies formed.

To generate stable Flp-In T-REx MDCKII cells containing Ruby-EB3, EB3 was excised from mCherry-EB3 and inserted into pcDNA5/FRT/TO-neo-Ruby (kindly provided by Dr. J. Pines) between BamHI and NotI sites to create the plasmid pcDNA5/FRT/TO-neo-Ruby-EB3. This was co-transfected with pOG44 (1:9) into MDCKII host cells, and proper recombination events were selected with 500 μg/ml G418 (InvivoGen). To induce Ruby-EB3 expression, cells were treated with 0.1 μg/ml tetracycline for 12 h before the assay. Stable cells were maintained in culture with tetracycline-free FBS (Clontech) to avoid background expression of the protein.

### Confocal Microscopy, Live Imaging, and Image Analysis

Confocal images were captured using either TCS SP5 or TCS SPE confocal Leica laser scanning systems equipped with DMI60000 and DM 2500 microscopes, respectively, and a HCX PL APO Lambda blue 63x 1.4 OIL objective at 22°C. Images correspond to maximal projections or optical sections according to each experiment. Mosaic images were automatically acquired with a Nikon eclipse Ti-e microscope controlled by NIS-Elements imaging software (Nikon) and using the scan-large-image tool.

For in vivo imaging experiments of AJ reassembly, cells expressing either control or CAP350 shRNAs (GFP-) were plated onto 35-mm glass-bottom dishes (IBIDI), transfected with peGFP-α-catenin and cultured in phenol red–free MEM. Two hours after EGTA addition, cells were washed, and transfected cells were identified and recorded at 37°C every 8 or 15 min for 5 or 12 h (as indicated) with an inverted microscope (DM16000; Leica) equipped with a camera (ORCA-ER; Hamamatsu Photonics) and using an HCX Plan Apochromat CS 63×/1.4 NA oil objective. For time-lapse experiments to visualise MT dynamics, Ruby-EB3-inducible MDCKII cells were plated three days after infection with shCAP lentivirus (GFP+). Noninfected Ruby-EB3-inducible MDCKII cells were also plated. The day after, Ruby-EB3 expression was induced for 12 h, and cells were recorded every 24 s for 288 s. For quantification of the area covered by isolated cells, cells were plated at a density of 10,000–20,000 cells/ml and analysed the day after seeding.

Image processing was carried out using the Leica (LAS) and Adobe Photoshop softwares. For presentation, whole images were adjusted for intensity level, contrast, and/or brightness. Quantification of cell surface was performed using the multiwave length cell-scoring module of Metamorph Offline software. Cyst diameter was calculated with ImageJ and the command Multimeasure. The cyst section with the widest transverse diameter was chosen for image analysis. For quantification of lumen formation, MDCKII cysts were phenotypically classified into three groups based on GP135 staining: single-lumen cyst (single central lumen), multiple-lumen cyst (≥two lumens) and aberrant cyst (cell aggregate without visible lumen). More than 200 cysts from randomly selected fields were examined under each condition. For quantification of centrosomal CAP350 fluorescence intensity, mosaic images were processed with Metamorph Offline software. After background subtraction, resulting objects were detected by using the “create regions around objects” command and objects’ intensities were estimated with the “region measurements” tool. For calculation of the number of EB3 comets, cells with comparable levels of Ruby-EB3 expression were selected and processed with the ImageJ software. Regions were created around selected cells, and image background fluorescence was corrected. The number of Ruby-EB3 comets per cell was calculated by using the “analyse particles” command. Frame 5 of each movie was selected for quantification. Between ten to fifteen cells of each group were quantified.

### Blocking Antibody Assay

To inhibit E-cadherin-mediated junction formation, we incubated MDCKII cells with 48 μg/ml anti-E-cadherin antibody (Monoclonal Anti-Uvomorulin/E-Cadherin clone DECMA-1, Sigma-Aldrich, U3254) in complete medium for 72 h; control experiments were run with cells incubated with a control IgG under the same conditions.

### Fish Stocks, Morpholino, and RNA Injections

Medaka fish (*O*. *latipes*) from the wild-type line Cab were kept as a closed stock. Embryos were staged as described [42]. A splicing morpholino (Gene Tools) directed against the medaka *cap350* exon5-intron5 junction was designed using as a template the medaka EST m010—E1_007 (Genebank: FM166216). The following morpholino was synthetized and injected: MCap350E5-I5: 5′-AATCCTTGAGACCAAATACCTTTAT-3′. Morpholino-induced splicing interference was monitored by RT-PCR in samples from wild-type and morpholino-injected embryos. To this end, total RNA was extracted from stage 15 embryos (TRIzol, Invitrogen), and RT reactions were performed (Super Script III, Invitrogen). The following primers were used in RT-PCR experiments:

Ctrl_ MCap350E5_fw: 5′- CAGAGGCAACATCTGGAGGAGG- 3′

Ctrl_ MCap350E7_rv: 5″- CGAGTCTATGGACCTTCCTAACTG- 3′

The vector *pCS2+*:*Lyn_tdTomato* was used as a template to synthesize capped RNA for the membrane-tagged tracer Lyn_tdTomato. Capped RNA was synthesize using the mMessage Machine Kit (Ambion) and column purified (Qiagen RNeasy). To monitor the success of the morpholino injection, the tracer was co-injected (50 ng/μl) with the morpholino (150 μM) into two-cell stage medaka embryos. Microinjected embryos were then examined under the fluorescence binocular (Olympus SZX16) and processed for immunofluorescence.

### Statistical analysis

Quantitative data are expressed as mean ± SD. Significant differences were evaluated by Student’s *t* test or ANOVA as appropriate (GraphPad Prism software).

## Supporting Information

S1 Data Excel spreadsheet containing the numerical data for Fig. 1, Fig. 5, Fig. 6D–6E, Fig. 6F, Fig. 7, Fig. 8C, and Fig. 8E. (XLS)

S1 Fig CAP350 specifically localises to cell–cell contacts. (**A**) IF analysis of CAP350 localisation in either nonextracted (left) or PHEM-Triton extracted (right) MCF10A cells. Hereafter, white arrows indicate the CTR. (**B**) WB analysis of MDCKII total extracts from noninfected cells (control) and cells infected with either control shm4 lentivirus or any of the three different CAP350 shRNA lentiviruses generated in this work (see Materials and Methods for details). GM130 was used as a loading control. (**C**) IF images of MDCKII cells under the same conditions as in (B) and labelled for CAP350, α-catenin, and α-tubulin. (**D**) MCF10A cells infected with shCAP lentivirus single labelled for CAP350 four days post-infection. (**E**) MDCKII cells infected with a mix of three lentiviruses (shCAP), fixed either 4 or 7 d post-infection and labelled with CAP350 and FOP antibodies. The boxed area marks the absence of CAP350 signal at cell–cell junctions, while white arrows indicate the remaining CAP350 signal at centrosomes. (**F**) MCF10A and NeuT cells labelled for CAP350. Enlarged image of the outlined area is shown (left). WB analysis of MCF10A and NeuT total extracts is shown at right. Bars = 10 μm.
(TIF)

S2 Fig Ectopic expression of either full-length CAP350 or the truncated mutant ΔN-CAP350. (**A**) Merged image of a MDCKII transfected with myc-CAP350 construct and labelled for myc and FOP. (**B**) MDCKII cells expressing myc-ΔN-CAP350 were stained with anti-myc and anti-α-catenin antibodies. (**C**) Defective cadherin-based cell–cell adhesion in the absence of junctional CAP350. Representative maximum projections of Z-stack images from either control (shm4, left) or CAP350-knockdown (shCAP, right) cells stained for E-cadherin and CAP350. Single labelling for E-cadherin and merged images are shown. (**D**) Determination of cell size by FACS analysis (counts versus forward scatter; FSC-H) of MDCKII cells infected with shCAP350 (shCAP) lentiviruses compared to those infected with control shm4 lentivirus. Data from three independent experiments are shown. Bars = 10 μm.
(TIF)

S3 Fig CAP350 is required for cadherin-based intercellular contact formation. (**A**) Live-cell imaging of MDCKII cells infected with either shm4 (left) or shCAP lentiviruses (right) and transfected with GFP-α-catenin. Cells were treated with 4 mM EGTA to disrupt cell–cell contacts. EGTA was washed out and cells allowed recovery time in complete culture media. Time after EGTA removal is shown. Yellow arrows indicate unstable cell-cell contacts in depleted cells compared to stable contacts in control cells at the same time points. (**B**) An overview of the procedure used to quantify the number of EB3 comets in time-lapse experiments shown in Fig. 7C and 7D. An original image of a Ruby-EB3–transfected MDCKII cell is shown at the left. Objects (red) obtained by thresholding image are shown in the middle panel, and final segmentation with estimated objects displayed in yellow and red are shown at right. Bars = 25 μm.
(TIF)

S4 Fig Proposed model for the CAP350/α-catenin mediated mechanism that regulates MT reorganisation during epithelial differentiation. CAP350 is recruited to AJs by interaction between its CAP2 and CAP4 domains and the VH1 domain of α-catenin. Once recruited to the AJ, CAP350 binds and could bundle MTs via its N-terminal domain. By linking E-cadherin, β-catenin, and α-catenin complexes at the plasma membrane with MTs, CAP350 may confer to cells the capacity to develop apico-basal MT arrays and to acquire columnar shape. In the absence of junction-located CAP350, transition from a radial mesenchymal MT array to an apico-basal epithelial one is blocked.
(TIF)

S1 Movie Calcium-induced AJ reassembly in MDCKII cells infected with shm4 lentivirus and transfected with GFP-α-catenin. In cells containing CAP350, α-catenin was detected at the cell surface 30 min after calcium addition. By 60 min, contacts between cells were re-formed.
(AVI)

S2 Movie Calcium-induced AJ reassembly in MDCKII cells infected with shCAP lentiviruses and transfected with GFP-α-catenin. Cells lacking CAP350 exhibited defective cadherin-based contact formation. α-catenin accumulated at spotlike junctions, but these primordial contacts seemed to be unstable and disappeared.
(AVI)

S3 Movie Calcium-induced AJ reassembly after EGTA treatment in MDCKII cells infected with shm4 lentivirus and transfected with GFP-α-catenin. Cells were recorded for 12 h after calcium addition.
(AVI)

S4 Movie Calcium-induced AJ reassembly after EGTA treatment in MDCKII cells infected with shCAP lentiviruses and transfected with GFP-α-catenin. Cells were recorded for 12 h after calcium addition.
(AVI)

S5 Movie Live-cell imaging of MT dynamics in subconfluent control MDCKII cells inducibly expressing Ruby-EB3. Cells were recorded 12 h after tetracycline addition.
(AVI)

S6 Movie Live-cell imaging of MT dynamics in subconfluent MDCKII cells infected with shCAP lentiviruses and inducibly expressing Ruby-EB3. Cells were recorded 12 h after tetracycline addition. In partially CAP350-depleted cells, both EB3 comets distribution and MT-nucleating activity of the CTR were indistinguishable from that of control cells (shown in S5 Movie).
(AVI)

S7 Movie Live-cell imaging of MT dynamics in polarised control MDCKII cells inducibly expressing Ruby-EB3. Cells were recorded 12 h after tetracycline addition. Four days after seeding, CTR nucleation activity of fully polarised cells became more difficult to visualise, and EB3 comets decreased in number and acquired a cortical distribution.
(AVI)

S8 Movie Live-cell imaging of MT dynamics in post-confluent MDCKII cells infected with shCAP lentiviruses and inducibly expressing Ruby-EB3. Cells were recorded 12 h after tetracycline addition. Four days after seeding, EB3 comets exhibited a radial distribution and arose from a perinuclear localised CTR that maintained a detectable MT nucleation activity.
(AVI)

## Abbreviations

AJ: adherens junction
Ab: antibody
cDNA: complementary DNA
co-IP: co-immunoprecipitation
CYLD: cylindromatosis
CAP-Gly: cytoskeleton-associated protein–glycine-rich
CTR: centrosome
DAPI: 4 ',6-diamino-2-fenilindol
EB1: end binding protein 1
EB3: end binding protein 3
E-cadherin: epithelial cadherin
EGTA: ethylene glycol tetraacetic acid
FACS: fluorescence-activated cell sorting
F-actin: filamentous actin
FSC-H: forward-scatter height
GFP: green fluorescent protein
GA: golgi apparatus
GTP: guanosine-5’-triphosphate
HDF: human dermal fibroblasts
IF: immunofluorescence
IgG: immunoglobulin G
MDCKII: Madin-Darby canine kidney II
MS: mass spectrometry
MT: microtubule
PCNT: pericentrin
PFA: paraformaldehyde
PHEM: Pipes-Hepes-EGTA-MgCl_2_ buffer
RT: room temperature
RT-PCR: reverse transcription polymerase chain reaction
SD: standard deviation
SILAC: stable isotope labelling by amino acids in cell culture
siRNA: small interfering RNA
shRNA: short hairpin RNA
TRM: TON1 Recruiting Motif
VH1: vinculin homology domain 1
WB: western blot
ZA: zonula adherens
ZO-1: zonula occludens protein 1
