# Supplementary figures and images for: Alpha-catenin-Dependent Recruitment of the Centrosomal Protein CAP350 to Adherens Junctions Allows Epithelial Cells to Acquire a Columnar Shape
Source: PLoS Biol. 2015 Mar 12;13(3):e1002087. doi: 10.1371/journal.pbio.1002087 (PMC4357431; doi:10.1371/journal.pbio.1002087)

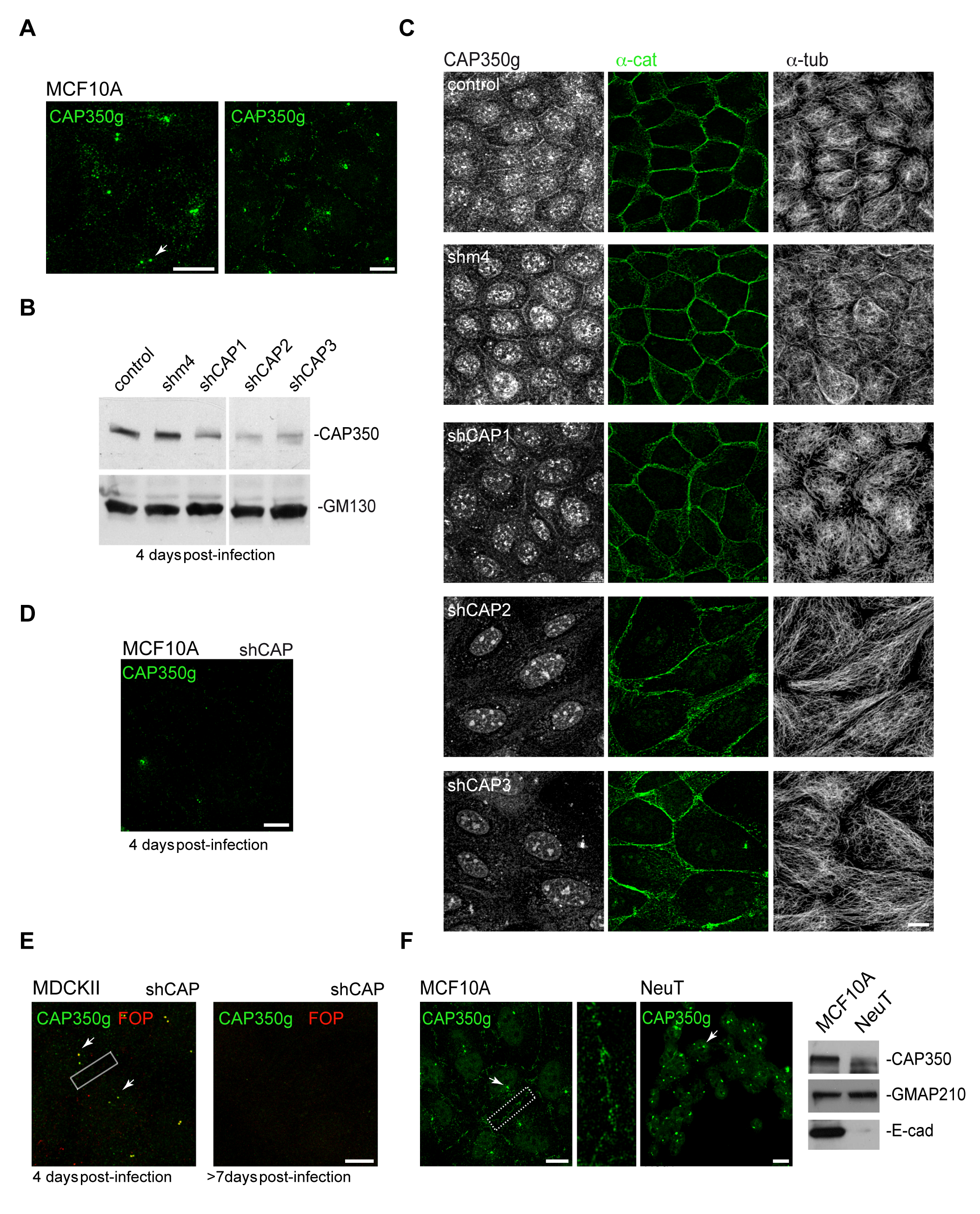

Supplement: S1 Fig — (A) IF analysis of CAP350 localisation in either nonextracted (left) or PHEM-Triton extracted (right) MCF10A cells. Hereafter, white arrows indicate the CTR. (B) WB analysis of MDCKII total extracts from noninfected cells (control) and cells infected with either control shm4 lentivirus or any of the three different CAP350 shRNA lentiviruses generated in this work (see Materials and Methods for details). GM130 was used as a loading control. (C) IF images of MDCKII cells under the same conditions as in (B) and labelled for CAP350, α-catenin, and α-tubulin. (D) MCF10A cells infected with shCAP lentivirus single labelled for CAP350 four days post-infection. (E) MDCKII cells infected with a mix of three lentiviruses (shCAP), fixed either 4 or 7 d post-infection and labelled with CAP350 and FOP antibodies. The boxed area marks the absence of CAP350 signal at cell–cell junctions, while white arrows indicate the remaining CAP350 signal at centrosomes. (F) MCF10A and NeuT cells labelled for CAP350. Enlarged image of the outlined area is shown (left). WB analysis of MCF10A and NeuT total extracts is shown at right. Bars = 10 μm. (TIF) [file pbio.1002087.s002.tif]

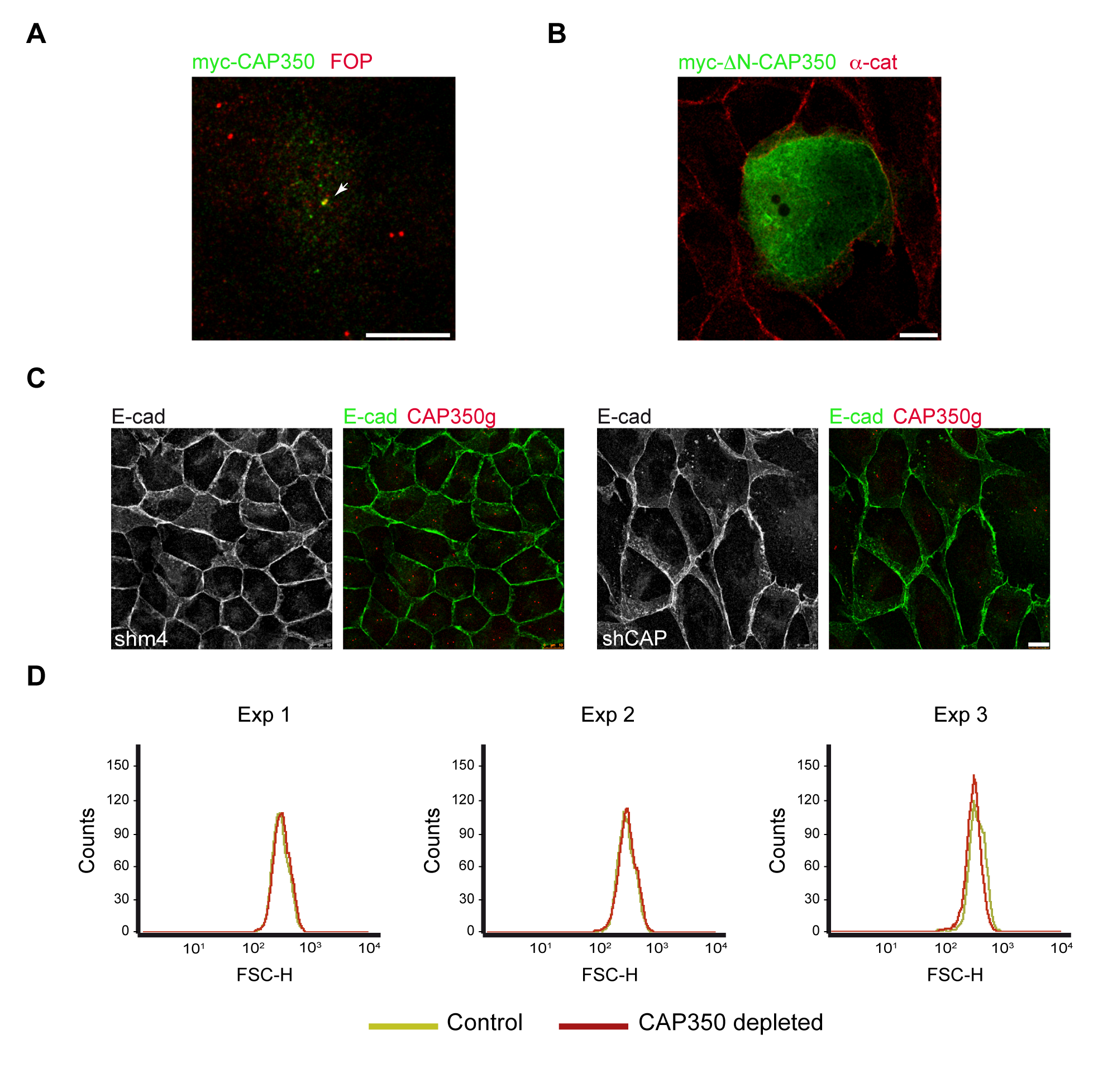

Supplement: S2 Fig — (A) Merged image of a MDCKII transfected with myc-CAP350 construct and labelled for myc and FOP. (B) MDCKII cells expressing myc-ΔN-CAP350 were stained with anti-myc and anti-α-catenin antibodies. (C) Defective cadherin-based cell–cell adhesion in the absence of junctional CAP350. Representative maximum projections of Z-stack images from either control (shm4, left) or CAP350-knockdown (shCAP, right) cells stained for E-cadherin and CAP350. Single labelling for E-cadherin and merged images are shown. (D) Determination of cell size by FACS analysis (counts versus forward scatter; FSC-H) of MDCKII cells infected with shCAP350 (shCAP) lentiviruses compared to those infected with control shm4 lentivirus. Data from three independent experiments are shown. Bars = 10 μm. (TIF) [file pbio.1002087.s003.tif]

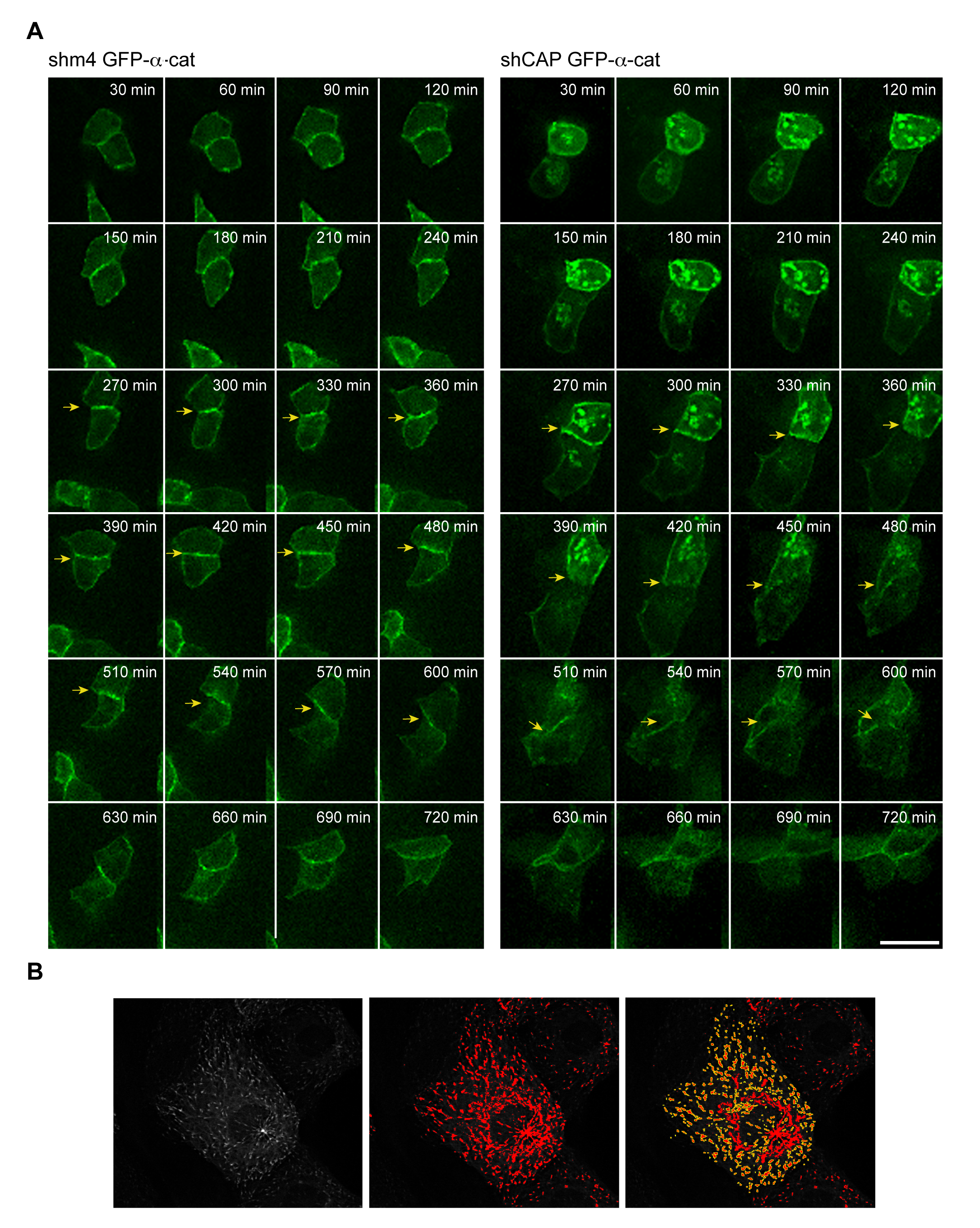

Supplement: S3 Fig — (A) Live-cell imaging of MDCKII cells infected with either shm4 (left) or shCAP lentiviruses (right) and transfected with GFP-α-catenin. Cells were treated with 4 mM EGTA to disrupt cell–cell contacts. EGTA was washed out and cells allowed recovery time in complete culture media. Time after EGTA removal is shown. Yellow arrows indicate unstable cell-cell contacts in depleted cells compared to stable contacts in control cells at the same time points. (B) An overview of the procedure used to quantify the number of EB3 comets in time-lapse experiments shown in Fig. 7C and 7D. An original image of a Ruby-EB3–transfected MDCKII cell is shown at the left. Objects (red) obtained by thresholding image are shown in the middle panel, and final segmentation with estimated objects displayed in yellow and red are shown at right. Bars = 25 μm. (TIF) [file pbio.1002087.s004.tif]

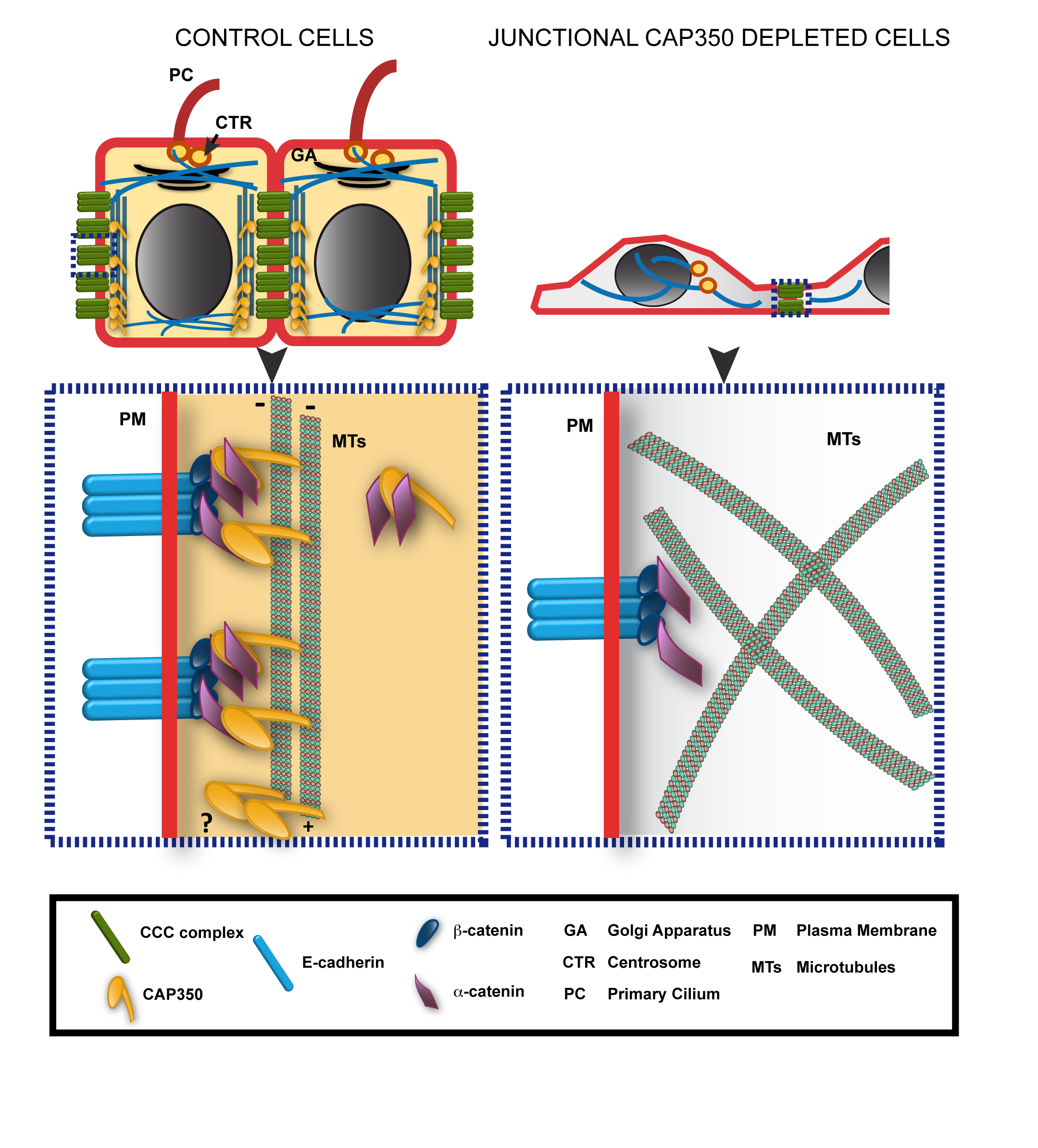

Supplement: S4 Fig — CAP350 is recruited to AJs by interaction between its CAP2 and CAP4 domains and the VH1 domain of α-catenin. Once recruited to the AJ, CAP350 binds and could bundle MTs via its N-terminal domain. By linking E-cadherin, β-catenin, and α-catenin complexes at the plasma membrane with MTs, CAP350 may confer to cells the capacity to develop apico-basal MT arrays and to acquire columnar shape. In the absence of junction-located CAP350, transition from a radial mesenchymal MT array to an apico-basal epithelial one is blocked. (TIF) [file pbio.1002087.s005.tif]
